# Avoidable under-5 mortality from congenital and genetic disorders: a frontier analysis of GBD 2023 identifying intervention targets

**DOI:** 10.3389/fpubh.2026.1886383

**Published:** 2026-07-28

**Authors:** Jingjing Ruan, Zewen Tao, Kaiye Zhang, Shaoran Wu, Ying Zhou, Xiaoxiao Yu, Hao Zhang, Guangyao Liu, Yue Zhang

**Affiliations:** 1 Liangzhu Laboratory, Department of Genetics and Metabolism, National Clinical Research Center for Children and Adolescents' Health and Diseases, Children's Hospital, Zhejiang University School of Medicine, Hangzhou, China; 2 Eye Center, The Second Affiliated Hospital, Zhejiang University School of Medicine, Hangzhou, China; 3 Zhejiang University-University of Edinburgh Institute (ZJU-UoE Institute), Zhejiang University School of Medicine, International Campus, Zhejiang University, Haining, China; 4 Huaihe Hospital of Henan University, Henan University, Kaifeng, China; 5 Department of Aesthetic Surgery, Jinan Yonghe Medical Aesthetic Clinic, Jinan, China

**Keywords:** congenital and genetic disorders, under-5 mortality, Global Burden of Disease, frontier analysis, avoidable mortality, health systems, global child health

## Abstract

**Introduction:**

Global under-5 mortality has fallen sharply since 1990, yet congenital and genetic disorders (ConGDs), spanning structural birth defects and inherited red-cell disorders (hemoglobinopathies and hemolytic anemias), have not previously been assessed as a composite burden. Effective interventions exist, but an implementation gap persists between availability and population-level delivery. We aimed to (1) quantify the temporal shift in the relative importance of ConGDs within under-5 mortality across 204 countries, and (2) apply frontier analysis to estimate avoidable ConGD mortality and identify intervention targets.

**Methods:**

Using GBD 2023, we aggregated 13 congenital and genetic causes (9 structural anomalies, 4 hemoglobinopathies and hemolytic anemias) for children under 5 from 1990 to 2023. We calculated age-standardized mortality rates (ASMR), proportional mortality ratios (PMR), and 95% uncertainty intervals (UI). Frontier analysis used log-transformed quantile regression (τ = 0.05) with natural cubic splines (*df* = 3) to estimate best-achievable mortality at each Socio-demographic Index (SDI) level.

**Results:**

ConGD ASMR fell 29.4% (105.70 [95% UI 72.44–153.23] to 74.64 [49.50–114.00] per 100,000), far slower than the 64.7% decline in communicable, maternal, neonatal, and nutritional (CMNN) diseases. PMR almost doubled (5.59% [3.78–8.20] to 10.28% [6.70–15.98]), reflecting differential decline rather than rising incidence. ConGDs moved from the 4th to the 2nd leading cause of under-5 death. ASMR correlated negatively with SDI (ρ = − 0.78); the PMR-SDI relationship was positive but is a compositional artifact of faster all-cause mortality decline at higher SDI. Mortality concentrated neonatally (0-6 days) for structural defects but peaked in early childhood for hemoglobinopathies. Frontier analysis estimated up to 251,423 (95% CI 201,692–289,940) potentially avoidable deaths in 2023 (52.4% of total), with efficiency gaps at every SDI level.

**Discussion:**

Nearly half of ConGD deaths in children under 5 are potentially avoidable. Three priority intervention targets emerge: (1) point-of-care newborn screening scale-up; (2) pediatric surgical capacity building for congenital heart defects; and (3) sickle cell chronic care programs that use proven therapies. A fourth, emerging opportunity lies in molecular diagnostics. Closing these gaps offers a high-impact route toward SDG 3.2.

## Introduction

Global under-5 deaths have fallen markedly over three decades, from 11.62 million (95% UI 11.48–11.77) in 1990 to 4.67 million (95% UI 4.59–4.75) in 2023 (1). Most of this decline came from expanded immunization, oral rehydration therapy, insecticide-treated bed nets, and improved nutritional supplementation (2, 3). As these causes recede, the remaining mortality increasingly involves conditions that require more complex, resource-intensive healthcare, including those with direct implications for global child health. Congenital and genetic disorders (ConGDs) are prominent among them (4–6). The March of Dimes estimated that 7.9 million children are born annually with serious congenital disorders (4), and the Every Newborn Action Plan identified birth defects as a priority requiring targeted interventions (7). Despite this recognition, most national child survival strategies remain focused on infectious and nutritional causes, and no global estimate has quantified the composite ConGD mortality burden. This is an implementation gap: effective interventions such as folic acid fortification, prenatal ultrasonography, neonatal cardiac surgery, newborn screening, and hydroxyurea therapy have demonstrated efficacy in high-resource settings but remain unavailable to most affected children globally.

The contribution of ConGDs to global child mortality has been studied only in fragments. Prior analyses focused on individual disease categories: congenital heart defects (8), neural tube defects (5), sickle cell disease (9, 10), or congenital birth defects as a single GBD category. None captured the aggregate burden. The GBD classification itself contributes to this fragmentation: congenital structural anomalies fall under “Congenital birth defects” at Level 2, hemoglobinopathies are classified under hematological conditions (11), and monogenic diseases such as cystic fibrosis are scattered across other categories. As a result, no study has assessed these disorders as a unified group, a gap with direct consequences for intervention design since intervention strategies differ between structural defects (requiring surgical correction) and hemoglobinopathies (requiring chronic disease management). While these conditions differ in cause and treatment, we group them for what they share in epidemiology and the health system. Three features recur. Each is present at birth, or fixed early by prenatal and genetic factors. Each has fallen behind as child-survival programs concentrated on infectious and nutritional causes. And each can be addressed, at least partly, with interventions that are already proven. The constraint is therefore one of implementation. Together these make the conditions a substantial remaining component of the epidemiological transition that is amenable to intervention, and that shared status is what makes it useful to treat them as a composite. Where treatments diverge, we keep them apart: the disease-specific frontiers below report surgical and chronic-care targets separately, in line with how GBD classifies these conditions.

Analytically, existing studies have relied on descriptive metrics such as average annual percentage change (AAPC) (12, 13). Frontier analysis, which measures the gap between observed and best-achievable outcomes at a given development level, was introduced for the GBD Healthcare Access and Quality (HAQ) Index (14) and applied as an explicit best-achievable frontier in its subsequent iteration (15); it has been used for healthcare-access and -quality indices but, to our knowledge, has not previously been applied to pediatric ConGDs. This approach has particular policy value: by quantifying the gap between what countries achieve and what is achievable at their development level, it moves beyond descriptive epidemiology to identify specific health system deficits amenable to intervention.

This study had two objectives. First, we quantified the temporal shift in the relative importance of ConGDs within under-5 mortality from 1990 to 2023 across 204 countries, using a composite measure that aggregates all congenital and genetic causes of death identifiable in the GBD framework. Second, we applied frontier analysis to estimate potentially avoidable mortality and to identify where efficiency gaps are largest, thereby identifying where the delivery of available interventions into health system practice could yield the greatest mortality reduction.

## Materials and methods

### Study design and data source

This global comparative assessment used data from the GBD Study 2023 (16–18). We extracted annual mortality data for children under 5 from 1990 to 2023 across 204 countries and territories. Institutional review board approval was not required because the study uses de-identified, publicly available data. This study adheres to the GATHER guidelines (Supplementary Table S1) (19).

### Disease selection and taxonomic justification

We aggregated 13 causes of death with distinct GBD identification codes into a composite mortality burden (Supplementary Table S2). These comprise 9 structural anomalies (congenital heart anomalies, neural tube defects, Down syndrome, other chromosomal abnormalities, orofacial clefts, digestive anomalies, urogenital anomalies, musculoskeletal/limb anomalies, and other congenital birth defects) and 4 conditions in the GBD hemoglobinopathies and hemolytic anemias category (sickle cell disorders, thalassemias, G6PD deficiency, and other hemoglobinopathies/hemolytic anemias). We refer to this second group as inherited red blood cell disorders (the GBD “hemoglobinopathies and hemolytic anemias” category): the hemoglobinopathies sickle cell disorders and thalassemias together with the red-cell enzymopathy G6PD deficiency. These are inherited disorders of the red blood cell and are reported separately from the structural birth defects throughout.

We use the term “congenital and genetic disorders” (ConGDs) to acknowledge that several included conditions have multifactorial etiologies rather than purely genetic causation. Neural tube defects (NTDs) are 50–70% preventable through folic acid fortification (5, 6, 20). Orofacial clefts have documented environmental risk factors, and G6PD deficiency manifests clinically through environmental triggers. Supplementary Table S3 presents the estimated modifiable fraction for each condition. Monogenic and metabolic conditions that GBD 2023 does not estimate as separable under-5 causes of death (cystic fibrosis, spinal muscular atrophy, organic acidemias, and other inborn errors of metabolism) were excluded, because GBD subsumes them within broader residual categories without a distinct under-5 mortality envelope; including them would require arbitrary apportionment and risk double-counting. This conservative approach minimizes misclassification but excludes some inherited conditions. Given these exclusions, the mortality estimates reported here represent a lower bound on the true burden.

### Metrics and statistical analysis

Primary outcomes included absolute deaths, ASMR (per 100,000 under-5 population — the GBD < 5 age-group mortality rate, which is computed against GBD's internal under-5 population denominator and is the conventional metric for cross-time comparison of under-5 burden), and PMR (the proportion of ConGD deaths relative to all-cause under-5 mortality). PMR is a compositional metric: when all-cause mortality declines faster than ConGD mortality, PMR will mechanically rise regardless of ConGD incidence trends. We therefore interpret PMR trends only as reflecting differential rates of decline, not changes in ConGD prevalence or severity. Age-specific mortality density (deaths per day) was calculated across developmental stages, and structural birth defects and hemoglobinopathies were analyzed separately to disentangle their age-specific patterns (Supplementary Table S4). As a complementary measure of disease burden incorporating both fatal and non-fatal consequences, we also report disability-adjusted life years (DALYs) and age-standardized DALY rates (per 100,000) for the same 13 ConGD causes; year-by-year DALY estimates 1990–2023 were extracted from the same GBD 2023 data release.

Given the non-linear relationship between SDI and burden metrics, we report Spearman rank correlations (ρ) rather than Pearson R. We do not report a PMR–SDI correlation coefficient. PMR and SDI are linked by construction. All-cause mortality is lower at higher SDI, and this mechanically inflates the proportional share of any slowly declining cause. A correlation coefficient would therefore quantify a compositional artifact rather than an epidemiological association. We return to this compositional relationship in the Discussion.

For frontier analysis, we employed quantile regression on log-transformed ASMR with natural cubic splines (3 degrees of freedom) to capture the non-linear SDI–mortality relationship:


$${\text{Q}}_{\tau }({\text{logASMR}}_{\text{i}}{\text{|SDI}}_{\text{i}})=f_{\tau }\left(SDI_{i}\right)$$

where *f*_τ_(·) is a natural cubic spline function. The 5th percentile (τ = 0.05) defined the frontier curve, representing the best-observed mortality at each SDI level (15). Natural splines were chosen over polynomial specifications because a quadratic model underfit the high-SDI range, placing the frontier well above the best-performing countries. Model validity was assessed through: (1) visual inspection of residual distribution (Supplementary Figure S1); (2) comparison of pseudo-*R*^2^ values across τ = 0.05 and τ = 0.10 specifications (Supplementary Table S5); (3) empirical coverage verification (percentage of countries below the frontier); and (4) leave-one-out cross-validation removing influential observations. Residual analysis showed no strong systematic trend across the SDI range, though modest residual structure remained in the lower tail (Supplementary Figure S1). Sensitivity analyses using τ = 0.10 were also conducted (Supplementary Figure S2). Model diagnostics are reported in Supplementary Table S5. The total number of potentially avoidable deaths was calculated as the sum across all 204 countries of max (observed – frontier-predicted deaths, 0); countries with observed mortality at or below the frontier (10 of 204 in 2023) contributed zero rather than negative values.

**Figure 1:**
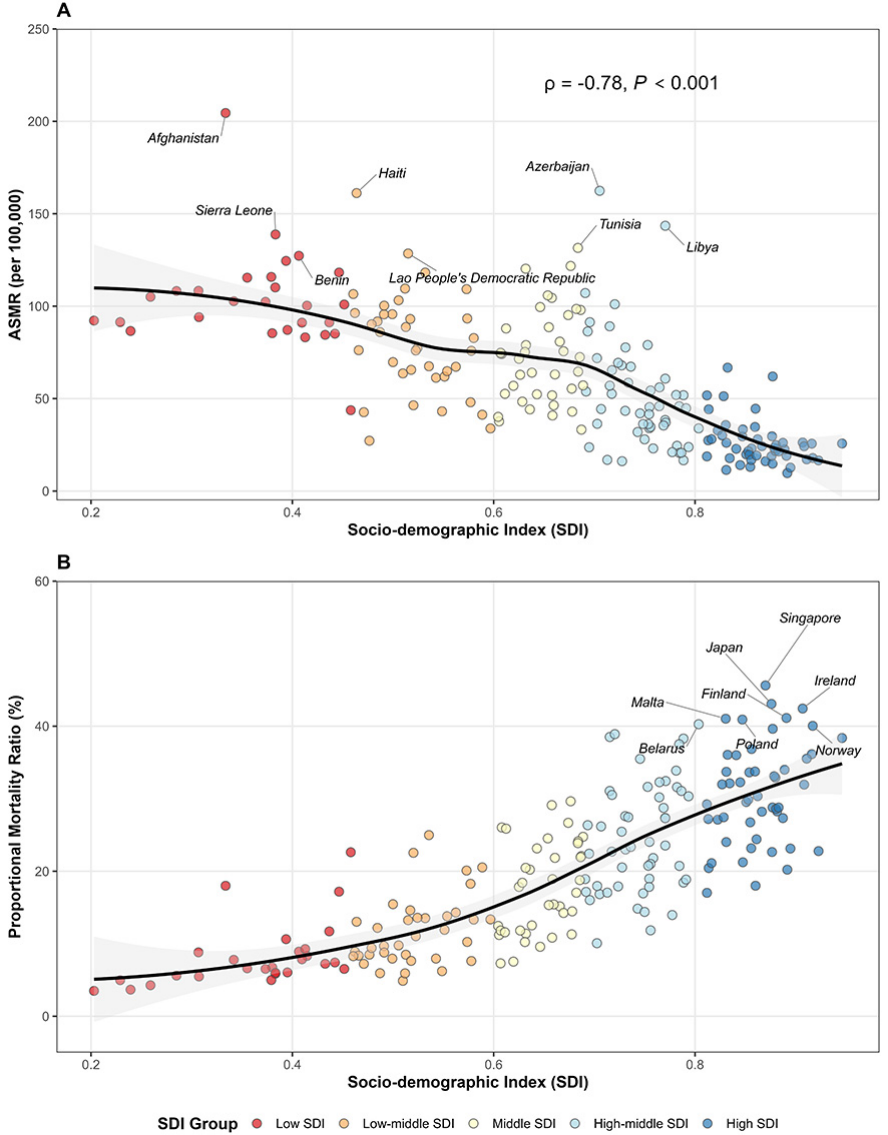
SDI vs. ConGD burden across 204 countries, 2023. **(A)** ASMR vs. SDI (Spearman ρ = −0.78); absolute mortality declines with development. **(B)** PMR vs. SDI; the higher PMR at higher SDI is a compositional artifact of faster all-cause mortality decline rather than increased ConGD risk (the correlation coefficient is not reported, as the two quantities are linked by construction; see Results). Countries color-coded by SDI quintile.

This frontier is an empirical, quantile-regression-based envelope rather than a data envelopment analysis (DEA) or a stochastic frontier analysis (SFA). DEA assumes a deterministic efficient frontier, and SFA parametrically separates inefficiency from noise; our approach instead estimates the conditional 5th percentile of observed log-ASMR given SDI, which makes no distributional assumption about inefficiency and is robust to outliers. We fixed the spline at 3 degrees of freedom a priori, the minimum needed to capture the documented non-linear SDI gradient (a steep decline at low SDI that flattens at high SDI) without the overfitting and reduced interpretability of higher-*df* specifications. A simple linear fit was rejected because at high SDI more than the nominal 5% of countries fell below it and its minimum lay above the best-performing country, underfitting the curvature evident in Figure 1A. The empirical coverage was close to nominal (4.9% vs. the 5% target); modest residual structure nonetheless remains in the lower tail (Supplementary Figure S1; see Limitations), and the parsimonious 3-*df* specification balances fit against the risk of overfitting the sparse lower quantile.

Countries highlighted in the frontier figures were selected algorithmically to avoid selection bias: within each SDI quintile, we identified the country with the largest positive residual (greatest efficiency gap) and the country nearest the frontier (smallest positive residual). The top-2 and bottom-2 countries globally by absolute efficiency gap were also included, yielding 8–12 representative countries spanning the full SDI and performance spectrum. Complete selection criteria and all top-10/bottom-10 countries are reported in Supplementary Table S6.

Analyses were performed using R version 4.4.3 (21) with the quantreg (Koenker R, version 6.1) and ggplot2 packages (21).

### Sex-stratified analysis

Given the X-linked inheritance of G6PD deficiency and documented sex differences in congenital heart defect prevalence, we performed sex-stratified analyses comparing male and female ASMR and temporal trends for both the composite ConGD burden and individual conditions. Results are reported in Supplementary Table S7 and Supplementary Figure S3.

### Trend quantification (EAPC)

For each location (Global, 21 GBD super-regions and regions, and—where longitudinal country data permit—each SDI quintile), we calculated the Estimated Annual Percentage Change (EAPC) in ConGD ASMR over 1990–2023 by fitting a log-linear regression on the natural logarithm of the annual ASMR against calendar year [ln (ASMR) = α + β·year + ε]. EAPC was derived as 100·[exp(β) – 1] with a 95% confidence interval from the standard error of β; trends were classified as decreasing (upper CI < 0), increasing (lower CI > 0), or stable (CI spans 0).

### Joinpoint trend analysis

To complement the single-segment EAPC estimate and detect inflection years in the 1990–2023 trajectory, we fitted a segmented (joinpoint) regression of ln (ASMR) on calendar year for Global and each of the 21 GBD regions using the R segmented package. Up to two joinpoints were permitted per location; the optimal number of joinpoints was selected by corrected Akaike information criterion (AICc) against the single-slope model. Within each segment we report the annual percentage change (APC) as 100 × [exp(β) – 1] with a 95% confidence interval from the segment slope's standard error. The average annual percentage change (AAPC) over the full period was computed as the segment-length–weighted mean of the APCs.

### Decomposition analysis

To separate the contribution of demographic change from epidemiological change to the 1990–2023 shift in absolute ConGD deaths, we performed a Kitagawa decomposition (22): ΔDeaths = ΔPopulation × mean (ASMR) + mean (Population) × ΔASMR. Under-5 population denominators were back-computed from the GBD-reported deaths and rate; the decomposition was applied at the Global level, in each GBD region, and within each SDI quintile to quantify the share of death-count change attributable to population growth vs. to mortality-rate improvement. The under-5 population denominator (P) was derived from GBD's mortality outputs as Number / Rate × 100,000 to maintain internal consistency with the mortality numerator.

### Predictive analysis

We projected Global ConGD ASMR—composite and separately for structural birth defects and for hemoglobinopathies—to 2035 using a Box-Jenkins ARIMA random-walk-with-drift model fitted to log-transformed annual ASMR via the forecast R package [Hyndman-Khandakar automatic model-order selection (23)]; the resulting ARIMA (0, 1, 1) specification on the log scale is the Frequentist counterpart of the Bayesian random-walk-2 (BAPC) projection used in single-age-band burden-forecast applications. Ninety-five-percent prediction intervals were computed from the model residual variance. The ARIMA (0, 1, 1) order was selected by the Hyndman-Khandakar algorithm, which minimizes the corrected Akaike information criterion (AICc) across candidate (*p, d, q*) models on the log scale; first-order differencing (*d* = 1) was indicated by the non-stationarity of the level series, and the residuals of the selected model were consistent with white noise (Ljung-Box *P* > 0.05), supporting the random-walk-with-drift specification.

### Bootstrap confidence interval for avoidable mortality

To quantify the uncertainty of the total avoidable ConGD deaths derived from the τ = 0.05 frontier, we performed a 1,000-replicate non-parametric country bootstrap: in each replicate we resampled countries with replacement, refit the natural-spline quantile regression, applied the resampled frontier to the original 204 countries, and summed the country-level positive residuals. The 95% confidence interval was taken as the 2.5th and 97.5th percentiles of the bootstrap distribution. This interval reflects sampling variability across the set of 204 countries treated as the bootstrap unit; it does not propagate the GBD-level mortality uncertainty intervals of the underlying cause-specific estimates.

### Use of AI tools

The authors used Claude (Anthropic) for manuscript copy-editing and minor debugging of R analysis scripts. All AI-assisted output was reviewed and verified by the authors, who take full responsibility for the content.

## Results

### Discordance between absolute and proportional burden

**Figure 2:**
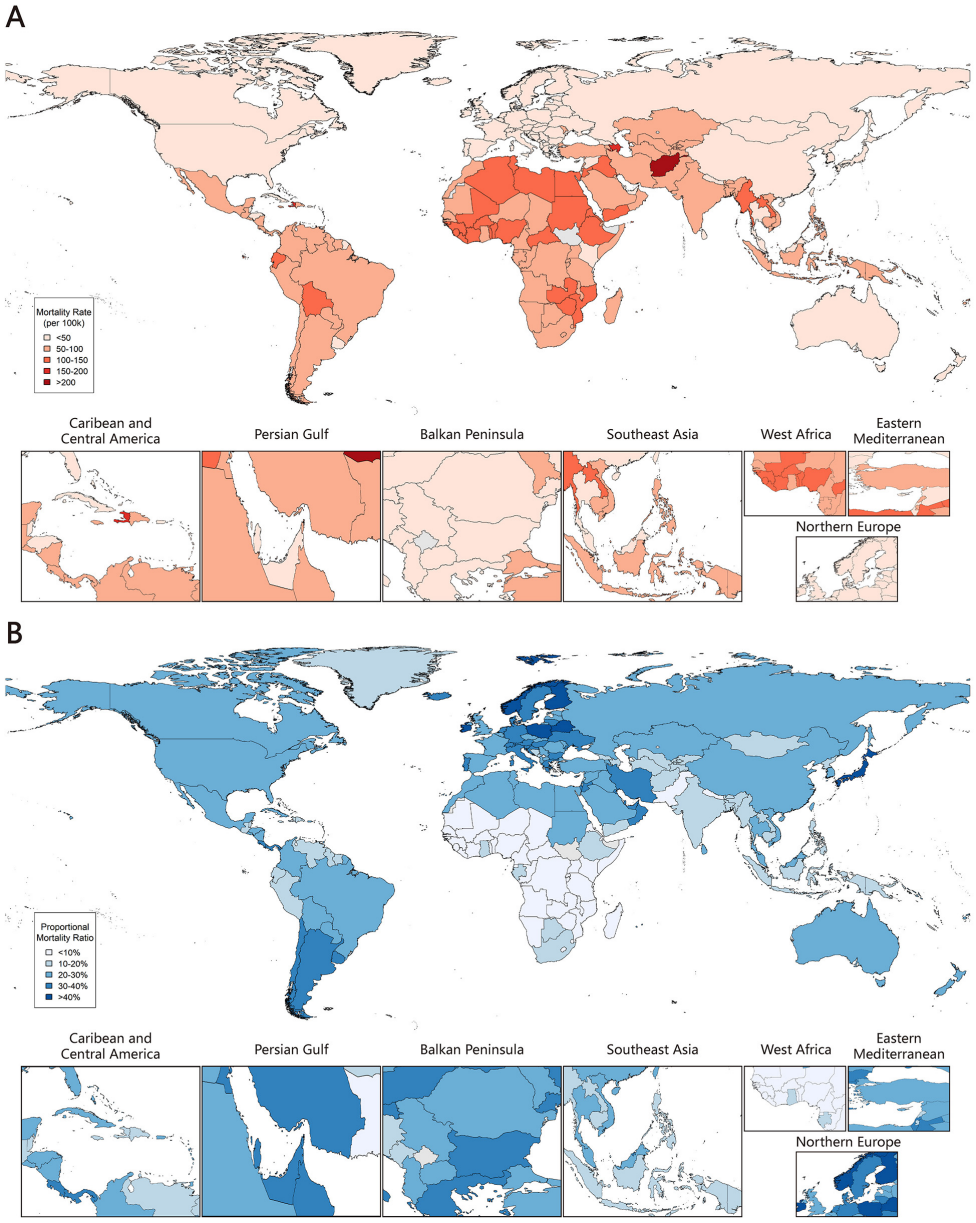
Global ConGD burden in children under 5, 2023. **(A)** Age-standardized mortality rate (ASMR, per 100,000) across 204 countries. **(B)** Proportional mortality ratio (PMR, %); high PMR in developed nations reflects lower all-cause mortality rather than higher ConGD incidence.

In 2023, the absolute burden (Figure 2A) peaked in low-SDI regions (ASMR 102.80 per 100,000), with the highest rates in sub-Saharan Africa and South Asia. The proportional burden (Figure 2B) showed the opposite pattern: it was highest in high-SDI regions (PMR 25.61%), exceeding 30% in several high-income subregions (Western Europe 30.45%, High-income Asia Pacific 37.28%). This discordance has direct policy implications: low-SDI countries need interventions that reduce absolute mortality (surgical capacity, newborn screening), while high-SDI countries must address ConGDs as the primary remaining target for further child survival gains.

### Global trends and diverging trajectories

**Figure 3:**
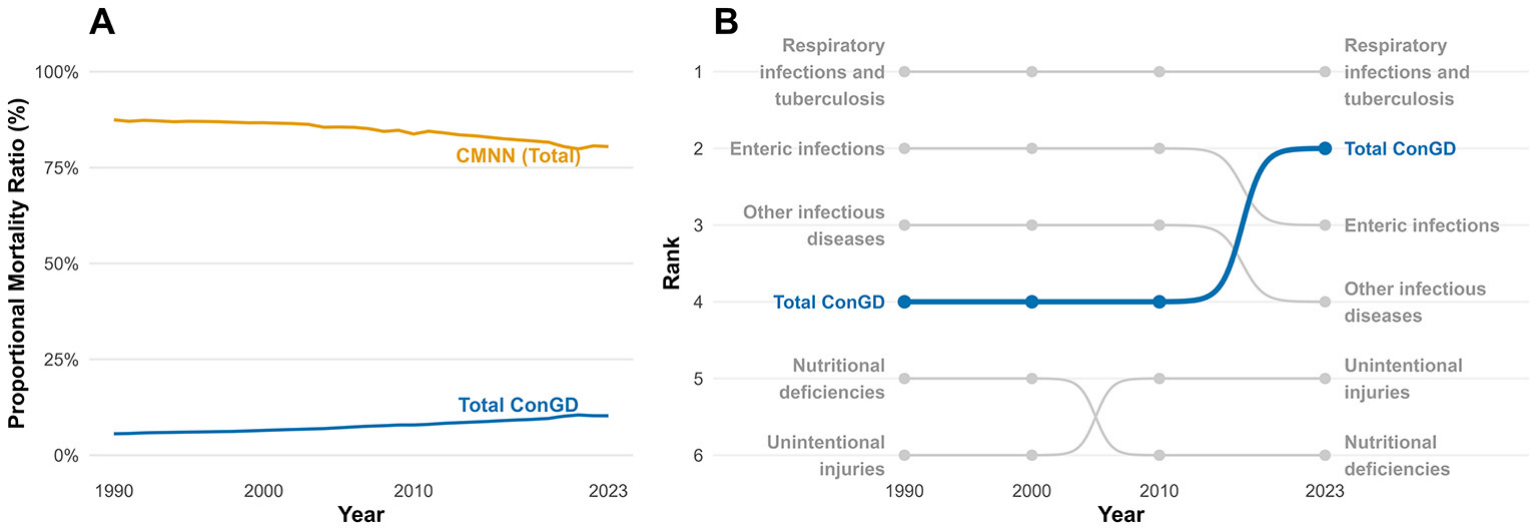
Diverging trends in proportional contribution and mortality ranking, 1990–2023. **(A)** PMR for ConGDs vs. CMNN diseases; the diverging trajectories illustrate how the faster decline of infectious mortality reveals the residual ConGD burden. **(B)** Bump chart of leading under-5 causes; ConGDs rise from rank 4 to rank 2 by 2023. The series plotted as “Total Genetic Disorders” in the figure denotes this composite ConGD burden; “genetic” is shorthand for the congenital-and-genetic composite and does not imply that every included condition is monogenic or present at birth (see Methods).

Between 1990 and 2023, the global ASMR for ConGDs in children under 5 declined by 29.4%, from 105.70 (95% UI 72.44–153.23) to 74.64 (49.50–114.00) per 100,000 (Table 1), corresponding to an absolute reduction in deaths from 649,326 (445,012–941,317) to 479,942 (318,314–733,052). This was markedly slower than the 64.7% ASMR decline in CMNN diseases over the same period [1,654 (1,615–1,691) to 584 (559–606) per 100,000]. Because other causes fell faster, the PMR nearly doubled from 5.59% (95% UI 3.78–8.20) to 10.28% (6.70–15.98) (Figure 3A). This rise stems from the faster decline of competing causes of death. By 2023, ConGDs had risen from the 4th to the 2nd leading cause of under-5 mortality among the seven major non-neonatal cause groups considered here (Figure 3B), surpassing enteric infections and other infectious diseases.

**Table 1:** Global and SDI-stratified ConGD mortality burden, 1990 and 2023.

	1990		2023		1990–2023	
Location	ASMR (95% UI)	PMR (%)	ASMR (95% UI)	PMR (%)	ConGD ASMR change (%)	CMNN ASMR change (%)
**Global**	105.70 (72.44–153.23)	5.59	74.64 (49.50–114.00)	10.28	−29.4	−64.7
High SDI	88.89 (67.67–115.52)	16.57	31.16 (26.12–40.20)	25.61	−65.0	−83.9
High-middle SDI	105.37 (78.70–145.13)	8.85	51.73 (41.07–66.86)	16.35	−50.9	−77.0
Middle SDI	104.28 (69.15–153.18)	6.95	79.24 (53.38–118.24)	17.15	−24.0	−74.7
Low-middle SDI	107.00 (64.93–171.75)	4.07	78.77 (52.70–124.67)	9.74	−26.4	−72.1
Low SDI	125.89 (65.80–219.06)	3.29	102.80 (59.26–171.85)	8.17	−18.3	−70.0

ASMR, age-standardized mortality rate per 100 000; PMR, proportional mortality ratio; UI, uncertainty interval; CMNN, communicable, maternal, neonatal, and nutritional diseases.

The CMNN ASMR Change column shows that the PMR increase is driven by the faster CMNN decline. The ConGD ASMR declined at every SDI level, with the gradient tracking health system capacity for surgical and diagnostic interventions. Regional-level data are in Supplementary Table S8. Regional heterogeneity was substantial: East Asia showed the steepest ConGD ASMR decline (−86.1%), paralleling its rapid economic development and health system expansion, while Southern Sub-Saharan Africa (+45.2%) and Central Asia (+1.5%) were the only regions where ConGD ASMR increased over the study period.

### SDI gradient and compositional artifact

ASMR correlated negatively with SDI (Spearman ρ = −0.78, *P* < 0.001; Figure 1A), confirming that absolute ConGD mortality declines with socioeconomic development. PMR was higher at higher SDI (Figure 1B). We do not report this as a correlation, because PMR and SDI are linked by construction: where all-cause mortality is lower (at higher SDI), the proportional share of any slowly declining cause is mechanically larger. The underlying compositional reasoning is developed in the Discussion rather than treated here as an epidemiological association. The compositional shift does, however, have practical consequences for policy: in high-SDI nations where infectious causes have been controlled, ConGDs are among the largest remaining contributors to pediatric death and thus the primary target for further mortality reduction. Country-level data for all 204 countries are reported in Supplementary Table S9.

### Compositional patterns

**Figure 4:**
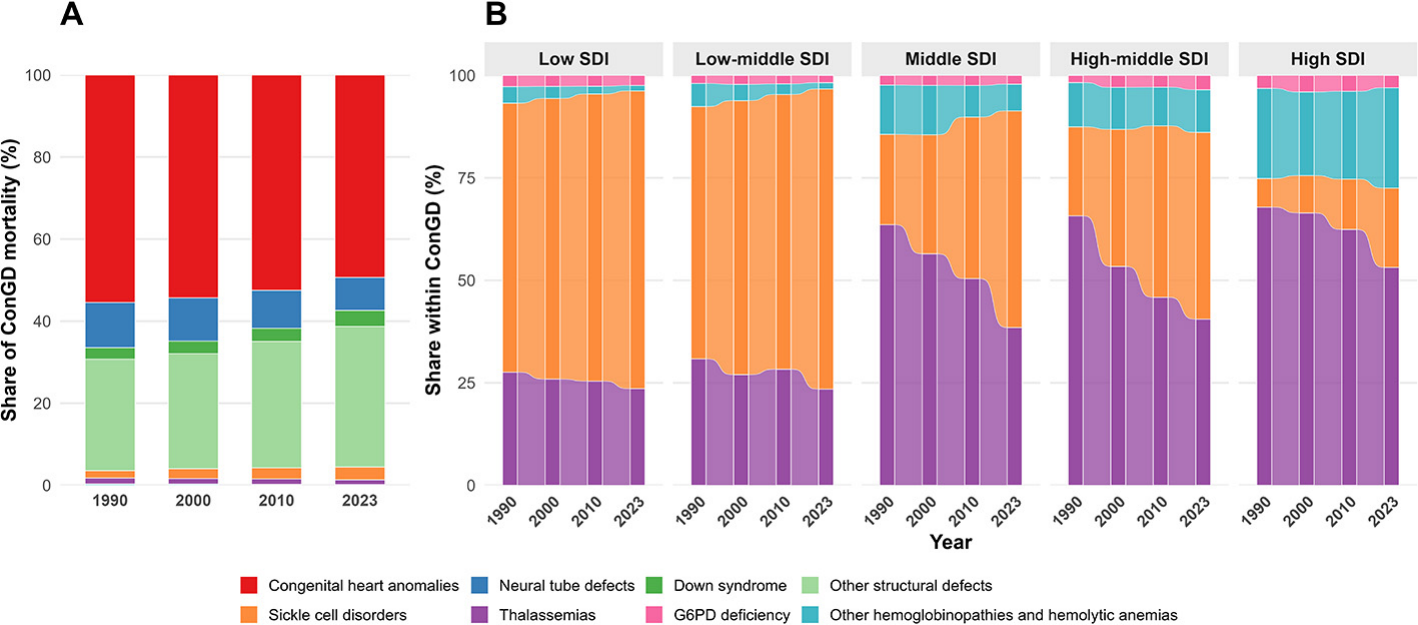
Compositional dynamics of ConGD mortality across SDI quintiles, 1990–2023. **(A)** Global proportional composition by cause at four time points; structural birth defects account for over 90%. **(B)** Cause-specific shares within each SDI quintile; hemoglobinopathies concentrate in low-SDI settings. Shares in both panels are expressed as a percentage of total ConGD mortality (the 13 included causes), not of all-cause under-5 mortality.

Congenital birth defects consistently accounted for over 90% of total ConGD mortality globally (Figure 4A). Including hemoglobinopathies increased the global ASMR by 4.64% (from 71.32 to 74.64 per 100,000) and the PMR by 0.46 percentage points relative to structural defects alone. This incremental contribution was concentrated in low and low-middle SDI quintiles, where sickle cell disorders accounted for a large share (Figure 4B). In high-SDI regions, hemoglobinopathy mortality was negligible. This compositional heterogeneity motivates SDI-stratified intervention strategies.

### Age-specific vulnerability

**Figure 5:**
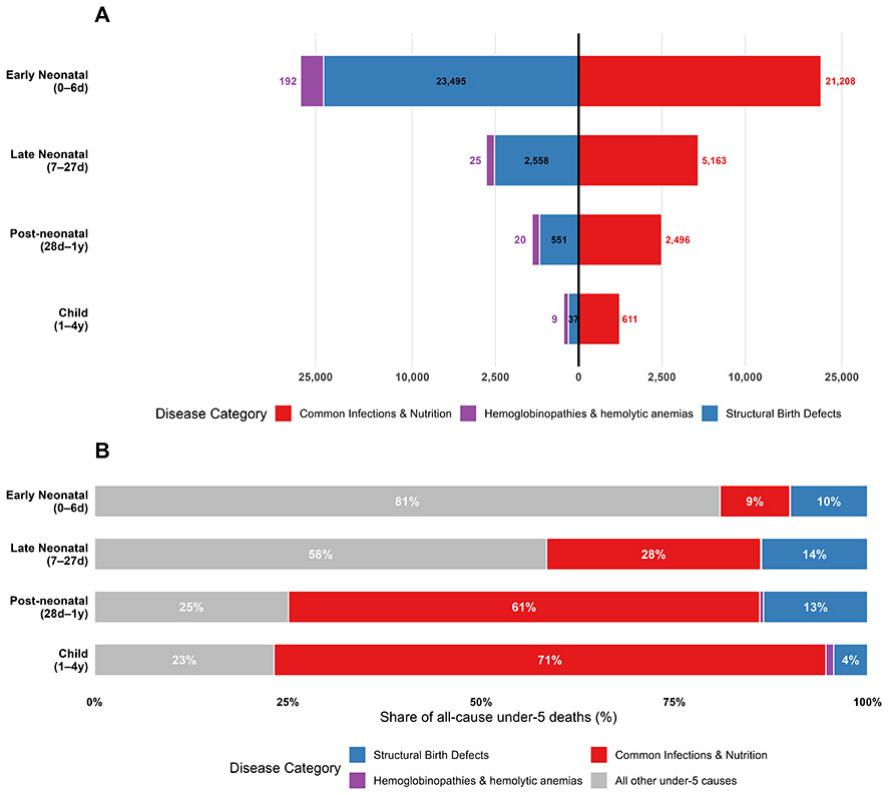
Age-specific ConGD mortality patterns, 2023. **(A)** Mortality density (deaths per day) for structural birth defects, hemoglobinopathies, and infections; structural defects peak neonatally, hemoglobinopathies in early childhood. **(B)** Age-specific proportional composition across categories; labels shown for segments ≥ 3%. In panel **(B)**, each cause group is shown as a share of all-cause under-5 mortality; the “all other under-5 causes” band comprises causes outside the ConGD and common infectious/nutritional groups (e.g., preterm birth, intrapartum complications, sepsis, injuries, and neoplasms). The hemoglobinopathy group denotes the GBD “hemoglobinopathies and hemolytic anemias” category (sickle cell disorders, thalassemias, G6PD deficiency, and other hemoglobinopathies/hemolytic anemias).

Mortality density analysis revealed distinct temporal windows for clinical intervention (Figure 5, Supplementary Table S4). For structural birth defects, density peaked at approximately 23,495 deaths per day during the early neonatal period (0–6 days) and fell more than 600-fold to roughly 37 deaths per day by early childhood (1–4 years). For hemoglobinopathies, the pattern was inverted: sickle cell deaths were minimal in the neonatal period and peaked in early childhood (2–4 years), consistent with the postnatal decline of fetal hemoglobin.

As a share of all-cause under-5 mortality, ConGDs accounted for about 10% of deaths in the first week of life and 5% at ages 1 to 4 years (Figure 5B); the remainder is dominated in the neonatal period by preterm birth, intrapartum complications, and sepsis, which fall in the “all other under-5 causes” band. When ConGDs are instead compared only with common infectious and nutritional causes, they made up 53% of that combined total in the first week and 7% at ages 1 to 4 years. The qualitative age gradient, with structural defects concentrated in the first week and hemoglobinopathies peaking later, holds under both framings. This narrow neonatal window for structural defects argues for early prenatal diagnostics and rapid newborn screening as priority interventions, while the later hemoglobinopathy peak points to the need for sustained chronic care programs. These are two distinct intervention modalities that require different health system capacities.

### Sex-specific patterns

Sex-stratified analysis showed clear differences for conditions with X-linked inheritance (Supplementary Table S7, Supplementary Figure S3). G6PD deficiency mortality had a strong male predominance (male-to-female ASMR ratio: 2.59), consistent with X-linked hemizygous expression in males. Thalassemias also exhibited a male excess (ratio 1.45). In contrast, sickle cell disorders showed a female excess (ratio 0.76); this is biologically unexpected for an autosomal recessive condition with equal genotype frequencies between sexes, and likely reflects sex-differential ascertainment, nutritional and infection co-morbidities affecting female under-5 survival, and structural assumptions of the GBD modeling framework rather than a true sex-linked biological mechanism. Overall, composite ConGD ASMR was 4% higher in males than females (M:F ratio 1.04). Temporal trends were parallel between sexes, with no evidence of sex-specific divergence over the study period. These sex-specific patterns are relevant to the design of screening strategies.

### Frontier analysis and efficiency gaps

**Figure 6:**
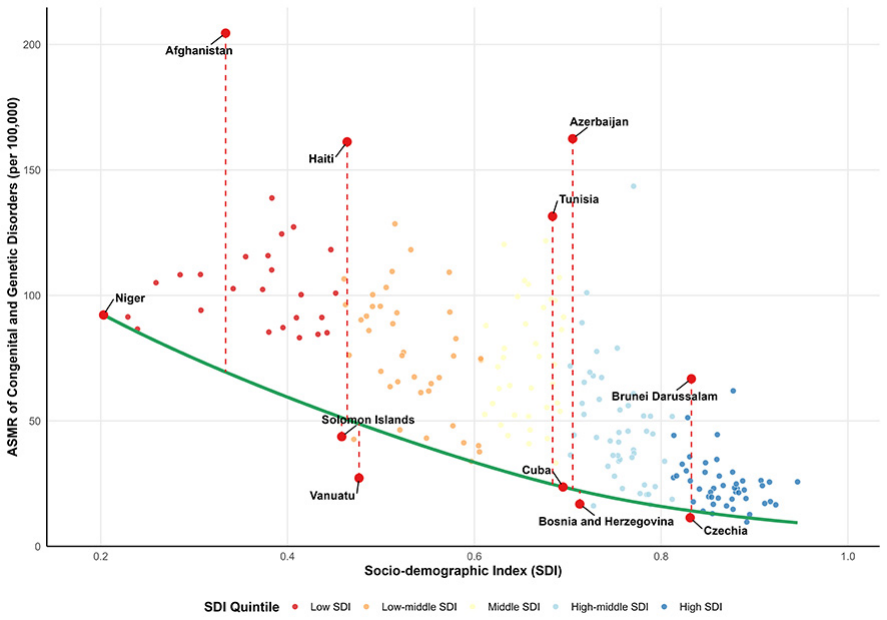
Frontier analysis identifying intervention targets, 2023. SDI vs. ASMR across 204 countries. Green curve: effective frontier (5th-percentile quantile regression), representing the best-achievable mortality at each development level. Annotated countries selected algorithmically by efficiency gap within each SDI quintile (criteria in Supplementary Table S6). Vertical distance to frontier quantifies potentially avoidable mortality, that is, the implementation gap between proven intervention efficacy and population-level delivery.

The fitted frontier curve (Supplementary Table S5; pseudo-*R*^2^ = 0.32; empirical coverage 4.9%, approximating the nominal 5%) traced a curvilinear decline in best-achievable ASMR across the SDI spectrum (Figure 6). The pseudo-*R*^2^ of 0.32 is modest but typical for τ = 0.05 quantile regression, which targets the lower tail of the distribution rather than the conditional mean and therefore optimizes a different loss function than ordinary least squares. Residuals showed no strong systematic pattern across SDI, with only modest residual structure remaining in the lower tail (Supplementary Figure S1), and results were robust to the choice of τ (τ = 0.10 yielded a similar frontier shape with pseudo-*R*^2^ = 0.37; Supplementary Figure S2). Summing efficiency gaps across all 204 countries, we estimate up to 251,423 potentially avoidable ConGD deaths globally in 2023 under the best-observed performance benchmark, equivalent to 52.4% of the country-aggregated observed ConGD mortality (479,556 deaths summed across 204 countries). This is an upper-bound point estimate that should be interpreted with two caveats: in high-SDI settings, a fraction of these “avoidable” cases would have been averted by selective termination following prenatal diagnosis rather than by postnatal clinical care; and the benchmark itself rests on the 5% best-performing countries, whose own outcomes may partly reflect prenatal screening capacity. Sensitivity analyses restricted to high-quality VR countries (Supplementary Table S10) and excluding high-consanguinity MENA countries (Supplementary Table S11) did not materially shift the frontier curve (the recomputed avoidable estimate for the same set of countries changed by less than 2%), indicating low structural sensitivity.

Among the top-10 countries by efficiency gap (Supplementary Table S6), Azerbaijan (gap 139.7 per 100,000), Afghanistan (135.1), Libya (125.7), Haiti (110.7), and Tunisia (106.8) showed the largest absolute gaps; seven of the top-10 countries were in the high-middle or middle SDI quintiles, indicating that efficiency gaps are not confined to the lowest-income settings. Large gaps persisted at all SDI levels. Countries annotated in Figure 6 were selected algorithmically as described in Methods.

### Disease-specific frontiers: disaggregating the composite gap

**Figure 7:**
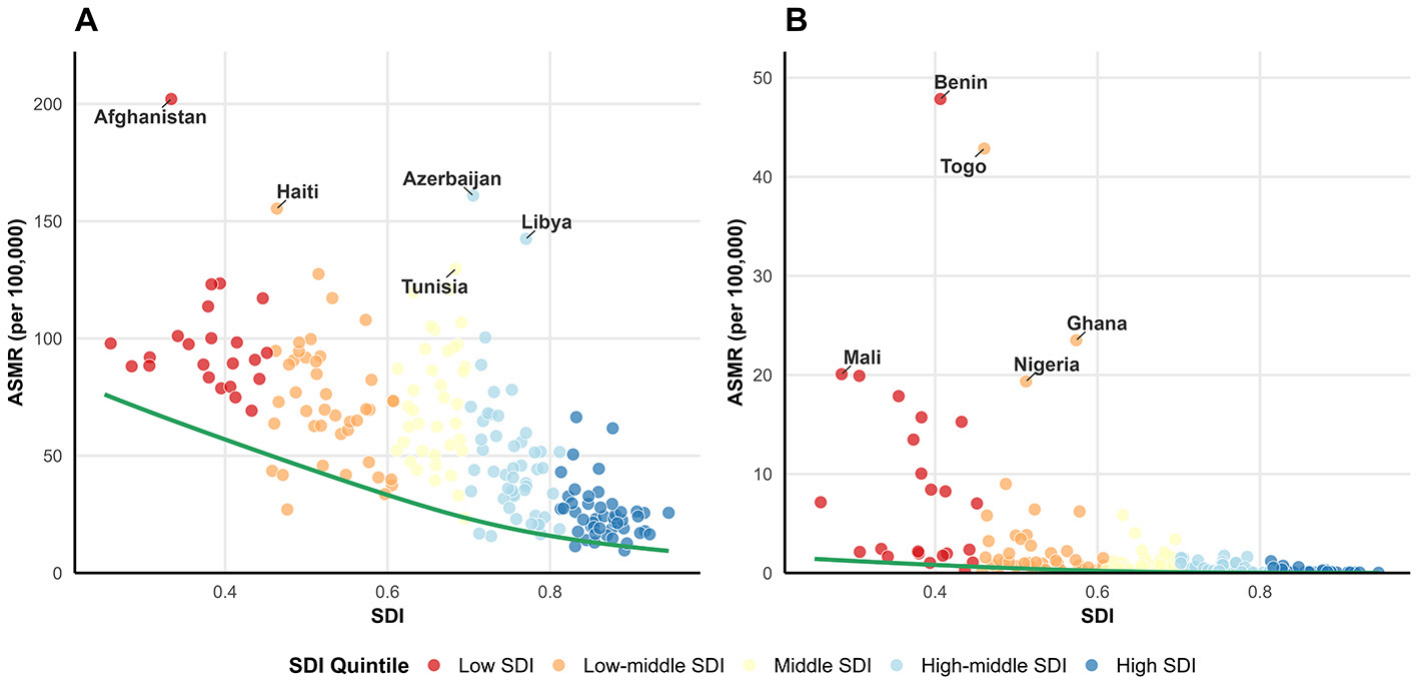
Disease-specific frontier analyses, 2023. **(A)** Structural birth defects only (9 causes); the curvilinear SDI gradient mirrors the composite frontier, identifying surgical-infrastructure gaps. **(B)** Hemoglobinopathies only (4 causes); the flatter frontier reflects allele-frequency anchoring in malaria-endemic regions, largely independent of SDI. Top-5 efficiency-gap countries labeled in each panel. Green curve: τ = 0.05 quantile regression with natural cubic spline (*df* = 3).

Because structural birth defects and hemoglobinopathies require fundamentally different interventions (surgical correction vs. chronic care), we fitted separate frontiers for each disease group (Figure 7). The structural-defect frontier preserved the curvilinear SDI gradient observed in the composite analysis, indicating that surgical capacity scales steeply with development and that countries distant from the frontier (e.g., Azerbaijan, Haiti, Afghanistan) face a predominantly surgical-infrastructure gap. In contrast, the hemoglobinopathy frontier was substantially flatter across the SDI spectrum, consistent with hemoglobinopathy burden being driven largely by allele frequency and malaria co-endemicity rather than by health system capacity: even some high-SDI malaria-origin populations retain elevated hemoglobinopathy mortality. This disaggregation has direct policy implications, since a single “efficiency gap” number can conflate two interventionally distinct targets, and country-level prioritization must identify whether a given nation's gap is dominated by surgical vs. chronic-care deficits. Country-level breakdowns of the disease-specific gaps are reported in Supplementary Table S6.

### EAPC trends across regions

The global EAPC of ConGD ASMR was −1.35% (95% CI −1.45, −1.26) over 1990–2023, indicating a slow, steady decline. Regional EAPC ranged from the steepest decrease in East Asia (−6.74%) to two regions showing significant increases: Southern Sub-Saharan Africa (+1.54%) and Central Asia (+0.40%). The regional ordering of EAPC closely mirrored the relative ASMR decline reported in Table 1 and confirms that the rising PMR reflects the much steeper concurrent CMNN decline. Detailed EAPC estimates for all 21 GBD regions are provided in Supplementary Table S12. Joinpoint regression on the same series identified two global inflections at 1996 and 2018 (joinpoint AAPC −1.03%; Supplementary Table S13); the most recent segment shows an attenuated decline post-2018 across most high-income and East Asian regions, and joinpoint detection further refines the regional pattern by exposing a post-2018 stall in several high-SDI Asian regions and a sustained increase in Southern Sub-Saharan Africa from 2005 onward.

### Decomposed contributions of demographics vs. epidemiology

**Figure 8:**
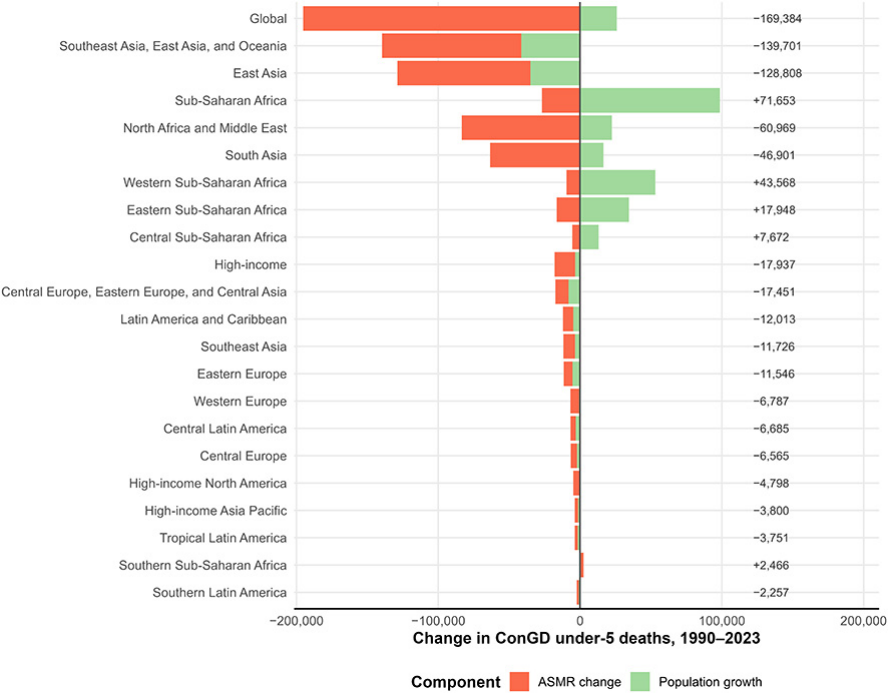
Kitagawa decomposition of the 1990–2023 change in absolute ConGD under-5 deaths into population-growth (green) and ASMR-change (red) components. Negative bars to the left of zero indicate components that reduced deaths.

Kitagawa decomposition of the 1990–2023 change in absolute ConGD U5 deaths showed that the global net reduction of 169,384 deaths was the sum of a population-growth effect of +25,916 (share −15.3%) and an opposing ASMR-improvement effect of −195,300 (share 115.3%). In other words, falling mortality rates would have prevented an additional 195,300 deaths had population size remained at 1990 levels; population growth masked the magnitude of epidemiological improvement. The pattern was most pronounced in low and low-middle SDI settings, where population growth offset more than half of the rate-based gains (Figure 8, Supplementary Table S14).

### Disability-adjusted life year (DALY) burden

The disability-adjusted life year (DALY) burden showed a parallel decline. Global ConGD DALYs in children under 5 fell from 60.14 million (95% UI 41.55–86.49) in 1990 to 44.99 million (30.12–67.98) in 2023, a 25.2% decline in absolute terms. The age-standardized DALY rate fell from 9,790.5 (6,763.3–14,078.7) to 6,995.8 (4,683.9–10,572.4) per 100,000, a 28.5% decline that closely mirrors the 29.4% drop in ASMR over the same period. The corresponding DALY EAPC was −1.31% (95% CI −1.40, −1.21), essentially identical to the ASMR EAPC of −1.35%, indicating that mortality reductions and disability reductions have advanced in lockstep and that the broader fatal-plus-non-fatal burden is shrinking at the same pace as deaths. SDI-stratified and regional DALY estimates are reported in Supplementary Table S15.

### Forecast to 2035

**Figure 9:**
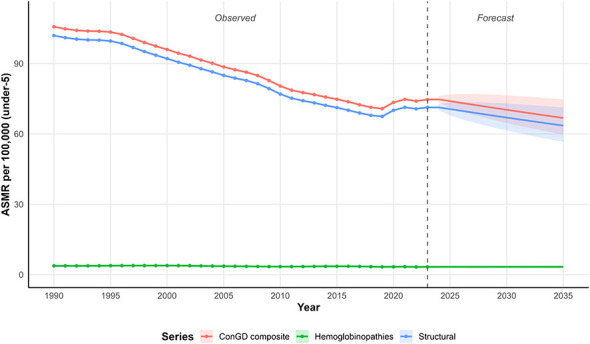
ARIMA forecast of Global under-5 ConGD ASMR per 100,000 to 2035. Three series shown: composite ConGD (sum of 13 causes), structural birth defects (9 causes), and hemoglobinopathies (4 causes). Observed years 1990–2023; forecast horizon 2024–2035 with 95% prediction intervals (shaded).

Under an ARIMA(0,1,1) random-walk-with-drift projection, the global ConGD U5 ASMR is expected to fall further from 74.64 per 100,000 in 2023 to 66.80 per 100,000 by 2035 (95% prediction interval 59.69–74.75; Figure 9). The forecast trajectory differs sharply by disease group: structural birth defects are projected to decline to 63.55 per 100,000, whereas hemoglobinopathies plateau at 3.33 per 100,000, reflecting the allele-frequency anchoring described above. Even under the optimistic continuation of historical decline, ConGDs will remain a leading composite cause of U5 mortality through 2035. Achieving SDG 3.2 will require action beyond the historical trajectory.

## Discussion

### Principal findings

This study is, to our knowledge, the first to treat ConGDs as a unified mortality burden among children under 5 using the most recent GBD 2023 data, and the first to apply frontier analysis to quantify avoidable ConGD mortality. The PMR of ConGDs nearly doubled from 1990 to 2023, moving them from the 4th to the 2nd leading cause of under-5 death. This shift in proportional share is an arithmetic consequence of faster CMNN decline (24, 25), not a biological increase in incidence. The ASMR of ConGDs declined at every SDI level (Table 1), and the PMR rise follows inevitably when the denominator (all-cause mortality) shrinks faster than the numerator (ConGD mortality).

Even so, the compositional shift matters for population health: in settings that have controlled infectious diseases, ConGDs are now the main target for further child survival gains. This finding aligns with the epidemiological transition framework (25), which predicts a shift from communicable to non-communicable causes as societies develop. However, unlike the classic transition model, which focuses on chronic adult diseases, the ConGD transition described here occurs within the first years of life and is driven by a failure to extend health system capacity to conditions requiring surgical and genetic expertise, leaving an implementation gap between available interventions and their delivery.

### Intervention targets identified by frontier analysis

The frontier analysis identified three systemic deficits amenable to intervention, presented below; an additional emerging molecular-diagnostic frontier is then discussed.

#### Diagnostic gap

Prenatal ultrasonography, non-invasive prenatal testing (NIPT), and newborn screening (NBS) remain unequal in their availability across countries (26, 27), a disparity now recognized in the WHO Essential Diagnostics List expansion. Extending these established diagnostic technologies to low-resource settings remains the central implementation challenge. However, successful models exist: Brazil's National Newborn Screening Program achieved >80% coverage within a decade of mandating heel-prick screening (28), showing that rapid NBS scale-up is feasible in middle-income settings when supported by legislative frameworks and centralized laboratory networks. Recent advances in point-of-care diagnostics, including dried blood spot screening and multiplexed immunoassays that do not require cold chains, offer promising pathways for extending NBS to the lowest-SDI settings (26). The estimated cost of NBS for sickle cell disease is US$1–5 per newborn in sub-Saharan Africa, making it one of the most cost-effective child health interventions available.

#### Surgical capacity gap

Structural anomalies account for over 90% of ConGD mortality, and survival depends on pediatric surgical capacity that is severely limited in low-SDI nations (29–31). The disease-specific frontier analysis (Figure 7A) confirmed that this gap is most pronounced for structural defects. The Lancet Commission on Global Surgery identified congenital anomaly repair as a bellwether procedure for surgical system development (29), providing a framework for transferring surgical expertise from high-SDI to low-SDI settings. Models already showing impact include: (1) hub-and-spoke cardiac surgery networks in India and Guatemala, where centralized high-volume centers serve as referral hubs for surrounding regions, achieving operative mortality rates approaching high-income benchmarks; (2) telemedicine-guided pre- and post-operative care, which extends specialist oversight without requiring physical presence; and (3) task-sharing models where trained general surgeons perform selected congenital procedures (e.g., cleft lip repair, colostomy for anorectal malformations) with outcomes comparable to specialist care in resource-constrained environments.

#### Chronic care gap

In sub-Saharan Africa, sickle cell disorders remain a major cause of avoidable mortality (32, 33) despite the availability of cost-effective interventions, including newborn screening, penicillin prophylaxis, and hydroxyurea (34). The REACH trial showed that hydroxyurea is safe and effective in sub-Saharan African children (34), yet implementation remains limited. This is a major implementation barrier between Phase III trial efficacy and real-world population-level delivery. The barriers are well-characterized: inconsistent drug supply chains, limited laboratory monitoring capacity, and insufficient trained workforce for chronic disease follow-up in primary care settings. The cost of hydroxyurea therapy is approximately US$50–100 per patient per year, far below the cost-effectiveness threshold for low-income countries, meaning the barrier is implementation infrastructure, not affordability. Newborn screening-to-treatment programs in sub-Saharan Africa provide a proof-of-concept that integrated screening-to-treatment pathways are achievable in low-SDI settings. At the frontier of disease-modifying therapy, the FDA approvals of exagamglogene autotemcel (exa-cel/Casgevy, the first CRISPR-Cas9 gene-editing therapy) (35) and lovotibeglogene autotemcel (lovo-cel/Lyfgenia) for SCD in December 2023 (36) demonstrate that curative options now exist; however, list prices of US$2.2–3.1 million per patient and reliance on autologous HSCT infrastructure place these therapies out of reach for the >75% of SCD births occurring in sub-Saharan Africa, which could widen the global access gap unless parallel access mechanisms are established.

#### A forward-looking diagnostic frontier

Beyond the three frontier-derived targets above, an emerging fourth opportunity lies in molecular diagnostics; while not directly quantifiable from mortality-based frontier analysis, it is increasingly relevant to the etiologically heterogeneous “other chromosomal” and “other congenital” categories. Targeted next-generation sequencing (NGS) panels and rapid whole-genome sequencing (rWGS) now achieve diagnostic yields of 40–60% in critically ill neonates with suspected genetic disease, with turnaround times of < 5 days, and have demonstrated direct changes in clinical management in 25–30% of cases (37). The BabySeq Project and the NSIGHT/NBSeq consortium have established the feasibility of population-level newborn genomic sequencing for actionable monogenic conditions (38, 39), and several high-income jurisdictions [England's Generation Study (40), New York's GUARDIAN study (41)] are now scaling this approach to hundreds of thousands of neonates. The policy implication for low- and middle-income countries is that the marginal cost of NGS has fallen below US$200 per sample, making regional sequencing hubs and cloud-based variant interpretation a feasible adjunct to existing newborn screening for the etiologic diagnosis of clinically opaque cases. Population-scale genomic newborn screening remains at the pilot stage.

These three intervention targets can be conceptualized within an implementation science framework. Using the Reach, Effectiveness, Adoption, Implementation, Maintenance (RE-AIM) framework (42), the primary barriers differ by target: for diagnostic scale-up, the bottleneck is Reach (coverage) and Implementation (laboratory infrastructure); for surgical capacity, it is Adoption (training and workforce) and Maintenance (sustainable referral systems); for chronic care, it is Implementation (supply chains) and Maintenance (long-term follow-up). This differential barrier profile implies that a single intervention strategy will not suffice; SDI-stratified implementation roadmaps are needed.

Efficiency gaps were not confined to low-SDI settings. Among the top-10 countries by efficiency gap (Supplementary Table S6), Azerbaijan, Libya, and Jordan are high-middle SDI countries (Tunisia is middle SDI), suggesting that development alone is insufficient and that country-specific health system factors, including consanguinity rates, prenatal care coverage, and pediatric surgical infrastructure, contribute substantially to the gap. Consanguinity in particular is a documented unmeasured confounder for several countries with the largest gaps: in North Africa, the Middle East, and Pakistan, first-cousin union rates of 20–50% (43) elevate the baseline incidence of autosomal recessive ConGDs above what an SDI-matched comparator would predict, meaning that part of the apparent “efficiency gap” reflects a higher biological floor rather than health-system underperformance. To probe this, we re-fitted the frontier on non-MENA countries (excluding 22 high-consanguinity countries from North Africa, the Middle East, Afghanistan, and Pakistan) and re-estimated avoidable mortality for the non-MENA set; the result was essentially unchanged (the non-MENA avoidable estimate changed by Δ < 0.1%; the frontier curve was unchanged), confirming that MENA observations do not anchor the frontier curve (Supplementary Table S11). However, MENA countries account for 58,937 avoidable deaths (23.4%) under the main analysis; this fraction should be interpreted as an upper bound, with the underlying biological component (consanguinity-driven baseline incidence) attributable to genetic counseling rather than clinical service delivery. This finding has direct implications: even countries with substantial health system capacity are failing to convert that capacity into optimal ConGD outcomes, pointing to specific implementation failures rather than resource limitations.

### Systematic bias of mortality-only framing

One caveat is that this analysis addresses mortality only, which biases comparisons across the SDI spectrum in a specific way. In high-SDI countries, advanced surgical capacity converts many formerly fatal ConGDs into chronic survivorship with ongoing morbidity; these children survive but accumulate years lived with disability (YLDs). The mortality-based PMR in high-SDI countries may therefore understate the true ConGD burden. In low-SDI countries where surgical capacity is absent, a larger fraction of ConGD cases prove fatal, inflating mortality-based metrics. Studies incorporating disability-adjusted life years (DALYs) would give a more complete picture and would likely show an even larger ConGD burden in high-SDI nations than mortality data alone suggest (8). We restrict the frontier analysis to mortality because non-fatal congenital anomaly registries in low- and middle-SDI settings rely heavily on covariate-based imputation within GBD, with substantially wider uncertainty than the death estimates anchored on vital registration data; incorporating YLD-derived DALYs into a τ = 0.05 quantile frontier would propagate that imputation uncertainty into the frontier curve and weaken the precision of the avoidable-mortality estimate that motivates this study.

Several included conditions also have modifiable risk factors (Supplementary Table S3), especially NTDs preventable through folic acid fortification, meaning some of this burden can be reduced through public health interventions without advanced surgical infrastructure, an important consideration for resource-limited settings.

### Prenatal screening and selective termination

A further consideration is the impact of prenatal screening on live birth prevalence. Many high-SDI countries have implemented widespread prenatal ultrasonography and NIPT. For conditions detectable in utero, this has led to high rates of selective termination, estimated at 67% for Down syndrome in the United States and exceeding 90% in several European countries (44). The resulting reduction in live birth prevalence means that the lower ASMR in high-SDI countries reflects both superior postnatal care and fewer affected live births. The frontier curve may therefore partly capture screening-and-termination capacity alongside treatment capacity. The GBD framework does not incorporate termination-adjusted prevalence, so this analysis cannot disentangle these pathways.

### Clinical and policy implications: an implementation roadmap

The implementation gap quantified in this study calls for a roadmap stratified by SDI context rather than a one-size-fits-all policy. We propose a three-tier framework aligned with the intervention targets identified above:

#### Tier 1, prevention (all SDI levels)

Universal folic acid fortification and periconceptional supplementation should be prioritized to prevent the modifiable fraction of NTDs, consistent with WHO recommendations (20). This is the most cost-effective intervention in the ConGD space: mandatory flour fortification costs less than US$0.10 per person per year and has reduced NTD prevalence by 30–70% where implemented. Countries without mandatory fortification policies, predominantly in sub-Saharan Africa and South Asia, should be prioritized.

#### Tier 2, early detection and triage (low and low-middle SDI)

Point-of-care NBS using dried blood spot technology should be incorporated into the WHO Package of Essential Non-communicable Disease Interventions, with priority for sickle cell disorders and critical congenital heart defects. Integration with existing immunization contact points (6-week DPT visit) maximizes reach with minimal additional infrastructure. The sex-specific differences identified in this study (Supplementary Table S7) may also inform screening strategies; for example, universal rather than selective G6PD screening in high-prevalence populations may be justified given the strong male predominance of symptomatic disease.

#### Tier 3, treatment scale-up (middle and high-middle SDI)

Pediatric surgical infrastructure needs strategic expansion, with congenital heart defect repair as the priority given its dominant contribution to ConGD mortality. The Lancet Commission on Global Surgery estimated that 143 million additional surgical procedures are needed annually in LMICs (29); our data suggest that CHD repair should be a bellwether procedure for tracking surgical capacity gains. In endemic settings, a targeted sickle cell care track integrating NBS, penicillin prophylaxis, and hydroxyurea access is needed (32). Task-sharing models that train non-specialist physicians and nurses in hydroxyurea initiation and monitoring can accelerate scale-up in settings with limited hematology workforce. Looking forward, equitable access pathways for the new SCD gene-editing therapies (exa-cel, lovo-cel) (35, 36) should be negotiated proactively through tiered pricing, voluntary licensing, and regional manufacturing partnerships, to prevent curative innovation from widening the global access gap. In parallel, integration of targeted NGS panels and rapid genome sequencing into tertiary neonatal units would enable etiologic diagnosis for the heterogeneous “other chromosomal” and “other congenital” categories that remain clinically opaque under current screening paradigms. For high-SDI countries, where infectious causes of under-5 mortality are largely controlled, the remaining priorities lie in equitable access to molecular diagnosis for clinically opaque syndromes, post-surgical survivorship care for children living with corrected congenital anomalies, and sustained investment in chronic management of inherited conditions in immigrant populations from high-prevalence regions.

### Strengths and limitations

Strengths include the 33-year temporal scope using the most recent GBD 2023 data, the first application of frontier analysis to pediatric ConGD epidemiology, the composite assessment integrating structural defects and hemoglobinopathies, and the systematic quantification of avoidable mortality. Model validation through residual diagnostics, sensitivity analyses, and leave-one-out cross-validation supports the robustness of frontier estimates.

This study has several limitations. GBD estimates for nations lacking robust vital registration (VR) rely on predictive covariates, and systematic ascertainment bias in low- and middle-income countries could in principle distort the frontier. To probe this, we conducted a sensitivity analysis re-fitting the frontier on the 71 countries with GBD data quality rating ≥ 4 stars (per the GBD 2019 data-quality appendix) and applying it globally (Supplementary Table S10). The total avoidable mortality estimate changed by less than 2% (251,423 → 248,559 deaths), confirming that our headline figure is robust to VR data quality. The high-quality VR frontier was *lower* in the lowest SDI range and *higher* in the middle SDI range than the main frontier, and these shifts offset globally; however, the quintile decomposition revealed that under the high-quality VR specification the share of avoidable deaths concentrated in low-SDI settings rose from 18% to 29%, strengthening rather than weakening the implementation gap argument in those settings. Kitagawa decomposition (Figure 8, Supplementary Table S14) uses under-5 population denominators derived from GBD mortality outputs to preserve internal consistency between numerator and denominator. Finally, the frontier-model residuals retain modest structure in the lower-SDI tail (Supplementary Figure S1), indicating that the τ = 0.05 quantile frontier does not capture all systematic variation; we retained the parsimonious specification rather than adding flexibility that would risk overfitting the sparse lower quantile, and the avoidable-mortality estimates should be read with this caveat.

The country-level ecological design precludes causal inference, and group-level efficiency gaps should not be interpreted as individual-level avoidable deaths (the ecological fallacy). The GBD framework lacks clinical severity grading, preventing stratification of lethal conditions (e.g., Trisomy 13/18) from surgically correctable ones. The composite frontier assumes homogeneous disease profiles across countries, though disease-specific frontiers partially address this (Figure 7). The mortality-only framing introduces a systematic bias as discussed above. Differential access to prenatal screening and selective termination across SDI levels confounds mortality comparisons (44). A sensitivity analysis restricted to the pre-COVID period (1990–2019) yielded essentially identical trend estimates, indicating that the pandemic did not materially distort the 33-year trajectories reported here (Supplementary Figure S4). Finally, the positive PMR–SDI correlation is a mathematical consequence of differential mortality decline rates and should not be read as a causal relationship between development and ConGD burden.

## Conclusion

Congenital and genetic disorders are now one of the largest remaining barriers to achieving SDG 3.2, and the implementation gap between proven interventions and their population-level implementation is the central modifiable factor. Using GBD 2023 data across 204 countries, this study demonstrates that the rising proportional contribution of ConGDs is an arithmetic consequence of success against infectious diseases, but the substantial efficiency gaps across the development spectrum reveal that this burden is not inevitable. Frontier analysis quantifies the magnitude of avoidable mortality and identifies three actionable intervention targets: (1) diagnostic scale-up through point-of-care newborn screening integrated into existing child health platforms, (2) surgical capacity building prioritizing congenital heart defect repair as the dominant contributor to ConGD deaths, and (3) chronic disease management through sickle cell care programs using proven, affordable therapies. Molecular diagnostics represent an emerging fourth, forward-looking opportunity. Some of this burden, particularly NTDs, is preventable through low-cost public health interventions already available. For the remaining burden, addressing the gap requires scaling up the delivery of established interventions rather than developing new ones; national health systems must incorporate these interventions into routine child health programmes. Future research should incorporate incidence data and age-period-cohort decomposition for a more comprehensive burden assessment, and implementation studies are urgently needed to evaluate context-adapted delivery models for the Intervention targets identified here.

## Data Availability

The original contributions presented in the study are included in the article/Supplementary material, further inquiries can be directed to the corresponding author.
