# Supplementary material for: Avoidable under-5 mortality from congenital and genetic disorders: a frontier analysis of GBD 2023 identifying intervention targets
Source: Front Public Health. 2026 Jul 28;14:1886383. doi: 10.3389/fpubh.2026.1886383 (PMC13457632; doi:10.3389/fpubh.2026.1886383)
Supplement: Supplementary file 1 [file Data_Sheet_1.pdf]

1. Supplementary Figures

Avoidable Under-5 Mortality from Congenital and Genetic Disorders: A Frontier Analysis of GBD 2023 Identifying Intervention Targets

1.1. Contents

Figures S1–S4
S1. Frontier residual diagnostics
S2. Frontier sensitivity
S3. Sex-stratified trends
S4. COVID-19 sensitivity analysis

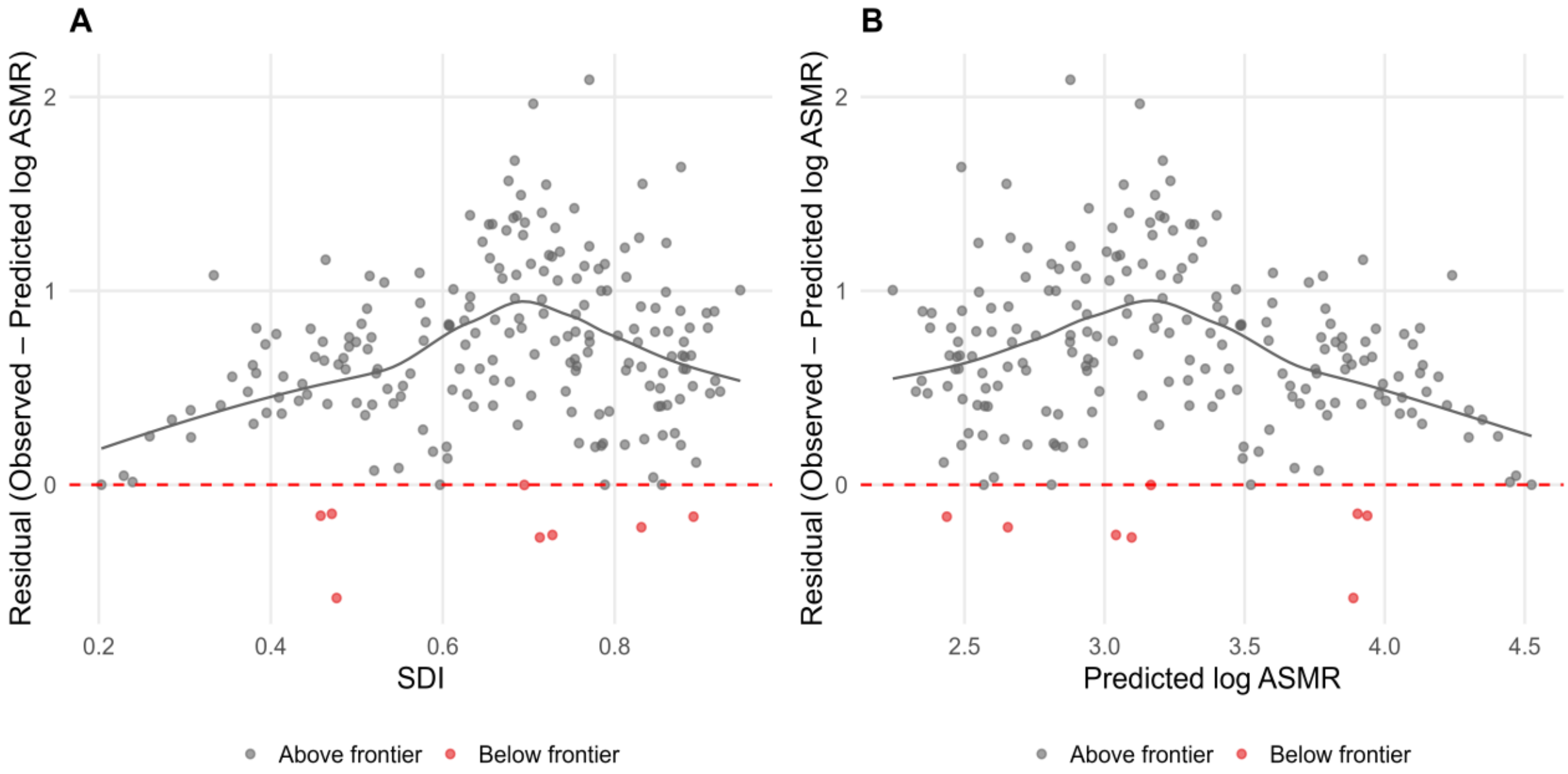

Figure S1. Frontier model residual diagnostics (2023). A, Residuals vs. SDI. B, Residuals vs. predicted values.

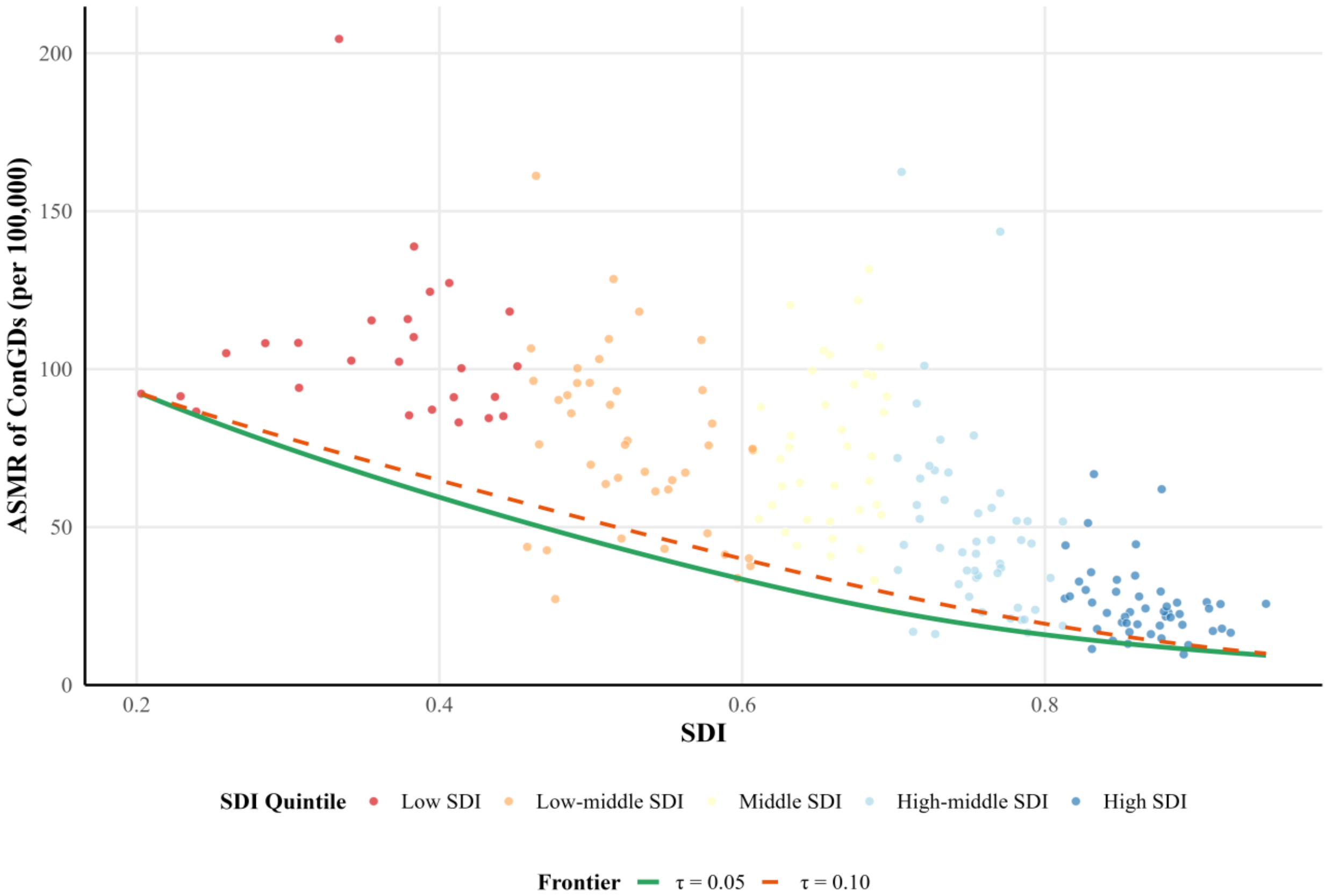

Figure S2. Sensitivity of frontier to quantile selection (2023). Solid green: 5th percentile (main). Dashed orange: 10th percentile.

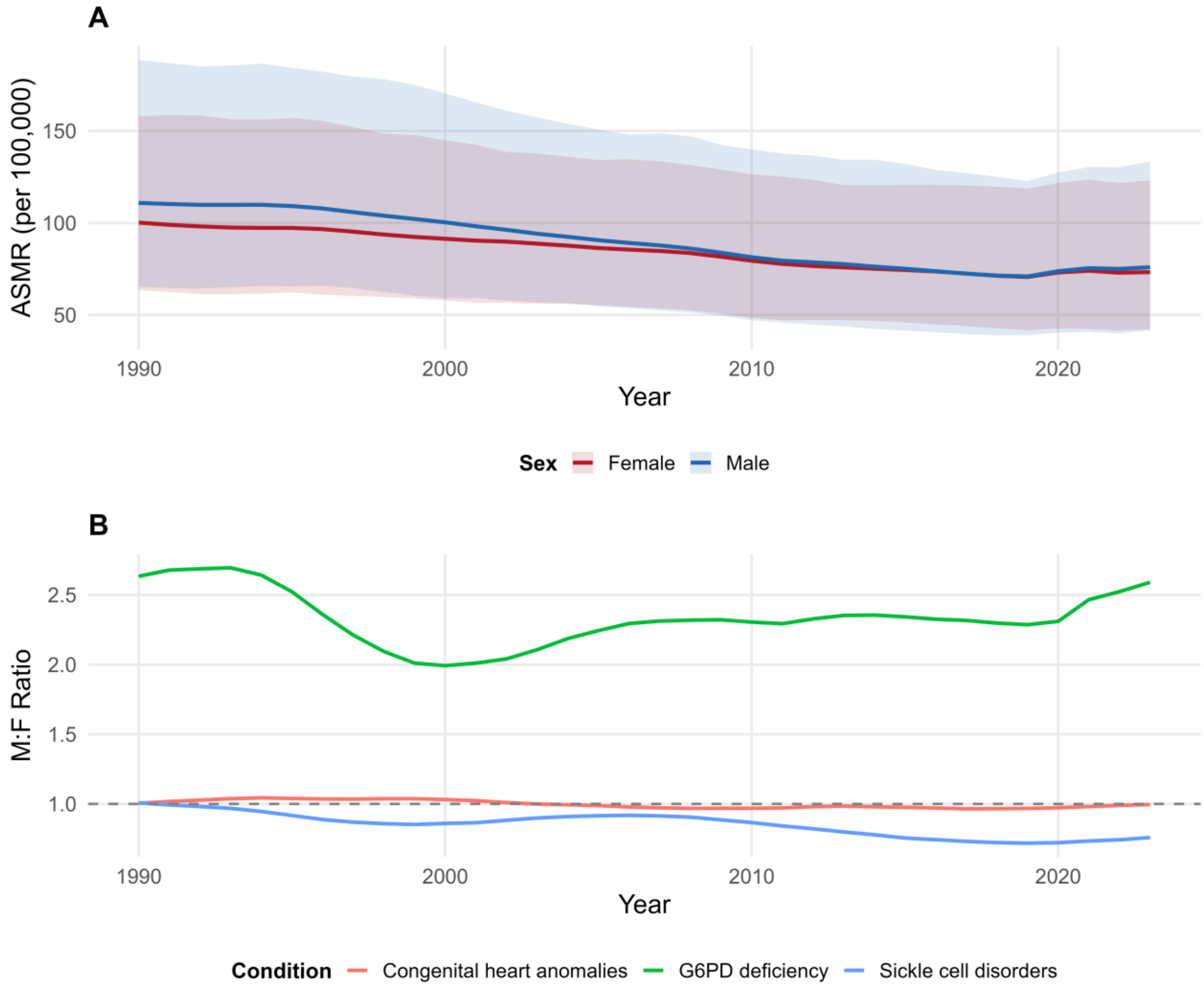

Figure S3. Sex-specific ConGD mortality trends (1990–2023). A, Composite ConGD ASMR by sex. B, Male-to-female ASMR ratio over time for key conditions.

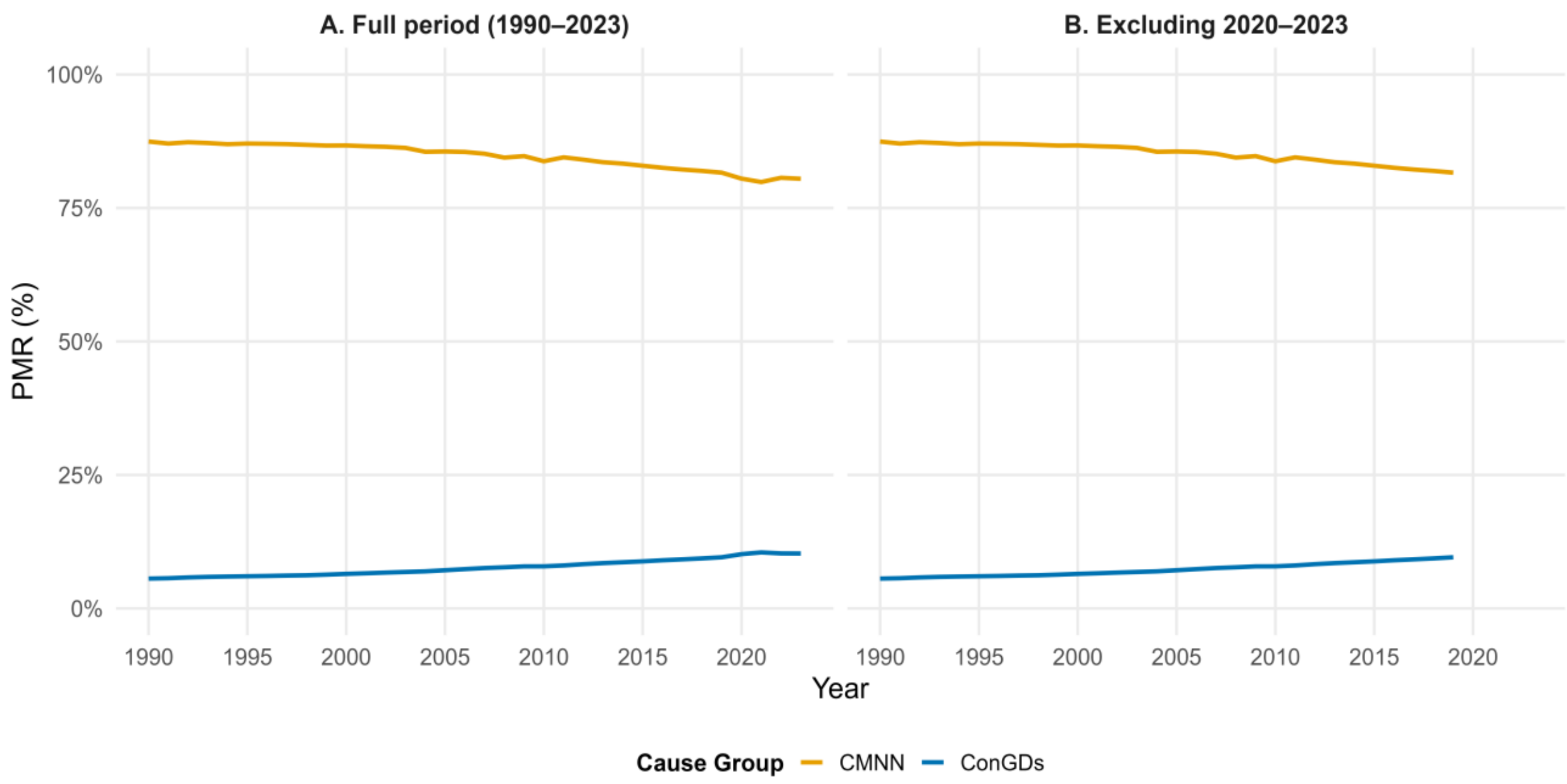

Figure S4. COVID-19 sensitivity: PMR trends with and without 2020–2023 data.
