# Supplementary material for: Avoidable under-5 mortality from congenital and genetic disorders: a frontier analysis of GBD 2023 identifying intervention targets
Source: Front Public Health. 2026 Jul 28;14:1886383. doi: 10.3389/fpubh.2026.1886383 (PMC13457632; doi:10.3389/fpubh.2026.1886383)
Supplement: Supplementary file 2 [file Supplementary_file_1.docx]

**Supplementary Tables**

*Avoidable Under-5 Mortality from Congenital and Genetic Disorders: A Frontier Analysis of GBD 2023 Identifying Intervention Targets*

**Contents**

**Tables S1–S14**

S1. GATHER reporting checklist

S2. Disease classification and GBD 2023 cause identifiers

S3. Estimated modifiable fraction (etiologic vs mortality)

S4. Age-specific mortality by cause (2023)

S5. Frontier model diagnostics (2023)

S6. Efficiency-gap rankings (top-10 / bottom-10)

S7. Sex-stratified ASMR (2023)

S8. Regional ConGD mortality burden (1990–2023)

S9. Country-level burden, all 204 countries

S10. High-quality VR sensitivity analysis

S11. MENA/consanguinity sensitivity analysis

S12. EAPC of ConGD ASMR, 1990–2023

S13. Joinpoint AAPC of ConGD ASMR, 1990–2023

S14. Kitagawa decomposition, 1990–2023

S15. DALY of ConGD in children under 5, 1990 vs 2023

Table S1. Guidelines for Accurate and Transparent Health Estimates Reporting (GATHER) checklist.

| **Section** | **Item** | **GATHER Checklist Item** | **Reported (Location in Manuscript)** |
| --- | --- | --- | --- |
| Objectives and funding | 1 | Define the indicator(s), populations (including age, sex, and geographic entities), and time period(s) for which estimates were made. | Methods: 13 ConGD causes, 204 countries, under-5 population, 1990–2023. |
|  | 2 | List the funding sources for the work. | Funding section. |
| Data inputs | 3 | Describe how the data were identified and how the data can be accessed. | Methods: GBD 2023 Results Tool (https://vizhub.healthdata.org/gbd-results/). |
|  | 4 | Specify the inclusion and exclusion criteria. Identify all ad hoc data exclusion criteria. | Methods: Table S1 lists inclusion criteria; no ad hoc exclusions applied. |
|  | 5 | Provide information on all included data sources and their main characteristics. | Methods: GBD 2023 database; Details in GBD 2023 capstone publications. |
|  | 6 | Identify and describe any categories of input data that have potentially important biases. | Discussion: Limitations (ascertainment bias in LMICs, mortality-only framing). |
|  | 7 | Describe and give sources for any other data inputs. | Methods: SDI covariate from GBD 2023 Socio-Demographic Index database. |
| Data analysis | 8 | Provide a conceptual overview of the data analysis method. | Methods: Descriptive epidemiology + quantile regression frontier analysis. |
|  | 9 | Provide a detailed description of all steps of the analysis, including mathematical formulae. | Methods: Quantile regression (τ = 0.05) with natural cubic splines (df = 3) on log-ASMR vs SDI. |
|  | 10 | Describe how candidate models were evaluated and how the final model(s) were selected. | Methods & Table S5: Model diagnostics, pseudo-R², knot sensitivity. |
|  | 11 | Provide the results of an evaluation of model performance, if done, as well as the results of any relevant sensitivity analysis. | Figures S1–S2; Tables S7, S11, S12: Sensitivity and cross-validation results. |
|  | 12 | Describe methods for calculating uncertainty of the estimates. | Methods: GBD uncertainty intervals propagated; bootstrap CI for frontier. |
|  | 13 | State how analytic or statistical uncertainty was factored into the evaluation of model performance, if done. | Table S5: Pseudo-R² and coverage probability reported. |
| Results and discussion | 14 | Provide published estimates in a file format from which data can be efficiently extracted. | Table S10: Full 204-country dataset (also provided as supplementary CSV). |
|  | 15 | Report a quantitative measure of the uncertainty of the estimates. | All estimates reported with 95% uncertainty intervals. |
|  | 16 | Interpret results in light of existing evidence. | Discussion: Interpreted in context of epidemiological transition, implementation gap. |
|  | 17 | Discuss limitations of the estimates. Include a discussion of any modelling assumptions or data limitations that affect interpretation. | Discussion: Limitations subsection addresses data quality, ecological fallacy, confounding. |
|  | 18 | State how the study was funded. | Funding section. |

Table S2. Congenital and genetic disorder classification and GBD 2023 cause identifiers.

| **Category** | **Specific Cause Name (GBD 2023)** | **GBD Cause ID** | **Inclusion Rationale** |
| --- | --- | --- | --- |
| Structural Birth Defects | Congenital heart anomalies | 643 | Largest proportion of fatal congenital defects globally. |
|  | Neural tube defects | 642 | Leading congenital cause of CNS-related neonatal death. |
|  | Down syndrome | 645 | Most common survivable chromosomal disorder. |
|  | Other chromosomal abnormalities | 648 | Includes Trisomy 13/18, Turner syndrome, etc. |
|  | Orofacial clefts | 644 | Common and typically surgically correctable anomalies. |
|  | Digestive congenital anomalies | 651 | Includes acute neonatal emergencies (e.g., esophageal atresia, diaphragmatic hernia). |
|  | Urogenital congenital anomalies | 650 | Includes renal agenesis and other genitourinary malformations. |
|  | Congenital musculoskeletal and limb anomalies | 649 | Includes clubfoot and other musculoskeletal deformities. |
|  | Other congenital birth defects | 652 | Other unspecified structural anomalies. |
| Hemoglobinopathies & Hemolytic Anemias | Sickle cell disorders | 615 | One of the most common monogenic diseases globally. |
|  | Thalassemias | 614 | Includes severe thalassemia major requiring regular transfusion. |
|  | G6PD deficiency | 616 | Leading enzymatic cause of neonatal kernicterus. |
|  | Other hemoglobinopathies and hemolytic anemias | 618 | Encompasses other rare hemolytic anemias. |

Table S3. Estimated modifiable fraction for included conditions: etiologic vs mortality modifiability.

| **Condition** | **Category** | **Etiologic Modifiability** | **Primary Etiologic Lever** | **Mortality Modifiability** | **Primary Mortality Lever** | **Key Reference** |
| --- | --- | --- | --- | --- | --- | --- |
| Neural tube defects | Structural | 50–70% | Folic acid fortification / supplementation | High (surgical repair) | Pediatric surgical capacity | Czeizel 1992; Blencowe 2018 |
| Orofacial clefts | Structural | 10–25% | Maternal smoking, alcohol, nutrition | High (surgical repair) | Pediatric surgical capacity | Mossey 2009 |
| Congenital heart anomalies | Structural | 5–10% | Maternal diabetes, rubella, obesity | High (surgical repair) | Pediatric cardiac surgical capacity | Jenkins 2007; Liu 2019 |
| Down syndrome | Structural | <5% | Advanced maternal age (screening-modifiable) | Moderate (cardiac surgery, supportive care) | Cardiac surgery + multidisciplinary follow-up | Natoli 2012 |
| Other chromosomal abnormalities | Structural | <5% | Advanced maternal age | Variable (condition-dependent) | Condition-specific | Natoli 2012 |
| Digestive congenital anomalies | Structural | <5% | Limited evidence | High (surgical repair) | Neonatal surgical capacity | — |
| Urogenital congenital anomalies | Structural | <5% | Endocrine disruptors (emerging evidence) | High (surgical repair) | Pediatric surgical capacity | Skakkebaek 2016 |
| Congenital musculoskeletal and limb anomalies | Structural | <5% | Limited evidence | Moderate to high (surgical / orthopaedic) | Orthopaedic / reconstructive capacity | — |
| Other congenital birth defects | Structural | Variable | Multiple / unknown | Variable | Condition-specific | — |
| Sickle cell disorders | Hemoglobinopathy | Low (Mendelian) | Carrier screening / genetic counselling | High (NBS + penicillin + hydroxyurea; gene therapy where accessible) | Newborn screening, penicillin prophylaxis, hydroxyurea | Piel 2017; Tshilolo 2019 (REACH) |
| Thalassemias | Hemoglobinopathy | Low (Mendelian) | Carrier screening / genetic counselling | High (transfusion + iron chelation; HSCT; gene therapy) | Safe transfusion, chelation, HSCT/gene therapy | Modell 2008 |
| G6PD deficiency | Hemoglobinopathy | Low (X-linked) | Carrier screening; avoidance of clinical triggers | High (avoidance of triggers; phototherapy; transfusion) | Newborn screening; trigger education | Cappellini 2008 |
| Other hemoglobinopathies and hemolytic anemias | Hemoglobinopathy | Low (Mendelian) | Carrier screening / genetic counselling | Moderate to high (transfusion + supportive care) | Transfusion access; etiologic workup | — |
| Etiologic modifiability = whether disease occurrence can be prevented; mortality modifiability = whether death from existing disease can be prevented. This distinction clarifies that hemoglobinopathies, although Mendelian in origin and therefore not preventable through public-health measures targeting incidence, have highly modifiable mortality through screening, prophylaxis, and chronic care. Etiologic modifiability refers to interventions that reduce live-birth prevalence (folic acid fortification, periconceptional care, premarital carrier screening). Mortality modifiability refers to interventions that reduce case-fatality among affected children (surgical repair, prophylaxis, disease-modifying therapy). Fractions are approximate and context-dependent. | | | | | | |

Table S4. Age-specific mortality (deaths, 95% UI) for congenital and genetic disorders in children under 5 years, Global, 2023.

| **Category** | **Cause** | **0-6 days** | **7-27 days** | **1-5 months** | **6-11 months** | **12-23 months** | **2-4 years** |
| --- | --- | --- | --- | --- | --- | --- | --- |
| Structural | Congenital heart anomalies | 62,970 (46,276–88,005) | 28,902 (22,265–37,272) | 78,960 (59,638–103,699) | 31,343 (23,374–41,391) | 15,281 (11,509–19,688) | 19,363 (14,500–24,715) |
|  | Congenital musculoskeletal and limb anomalies | 3,975 (2,460–6,450) | 732 (459–1,192) | 1,519 (944–2,425) | 719 (432–1,136) | 350 (219–553) | 395 (248–651) |
|  | Digestive congenital anomalies | 25,479 (16,503–36,802) | 9,309 (5,929–13,259) | 17,632 (10,173–26,953) | 7,060 (4,070–10,943) | 2,235 (1,302–3,627) | 2,237 (1,340–3,487) |
|  | Down syndrome | 3,011 (1,567–5,069) | 1,280 (727–2,109) | 6,043 (3,295–9,523) | 4,200 (2,311–6,919) | 2,111 (1,208–3,486) | 2,157 (1,240–3,368) |
|  | Neural tube defects | 22,752 (13,554–36,830) | 3,094 (1,811–5,630) | 6,839 (3,531–12,861) | 2,618 (1,410–4,864) | 1,202 (647–2,192) | 2,004 (1,161–3,638) |
|  | Orofacial clefts | 1,110 (233–3,214) | 387 (89–1,224) | 1,203 (283–3,609) | 398 (86–1,298) | 109 (27–375) | 66 (14–242) |
|  | Other chromosomal abnormalities | 7,734 (5,049–11,962) | 2,754 (1,784–4,497) | 5,900 (3,692–9,837) | 1,503 (923–2,504) | 739 (484–1,149) | 825 (537–1,313) |
|  | Other congenital birth defects | 29,594 (14,713–56,076) | 5,739 (2,903–11,681) | 12,520 (6,246–25,354) | 4,879 (2,423–10,046) | 2,189 (1,324–3,819) | 2,814 (1,688–5,058) |
|  | Urogenital congenital anomalies | 7,843 (3,907–15,057) | 1,516 (747–3,021) | 1,870 (713–5,030) | 510 (191–1,281) | 296 (116–651) | 368 (129–904) |
|  | **Structural Total** | **164,468 (104,262–259,465)** | **53,714 (36,715–79,885)** | **132,485 (88,517–199,291)** | **53,230 (35,220–80,382)** | **24,512 (16,836–35,540)** | **30,229 (20,859–43,376)** |
| Hemoglobinopathy | G6PD deficiency | 46 (18–100) | 20 (9–41) | 147 (71–278) | 63 (29–119) | 73 (36–127) | 143 (75–240) |
|  | Other hemoglobinopathies and hemolytic anemias | 108 (53–181) | 21 (15–32) | 139 (99–201) | 58 (45–75) | 65 (53–79) | 96 (83–110) |
|  | Sickle cell disorders | 699 (183–2,501) | 248 (92–694) | 2,217 (993–4,876) | 1,642 (685–4,007) | 3,027 (1,493–6,492) | 7,095 (3,565–15,115) |
|  | Thalassemias | 491 (209–1,037) | 227 (120–399) | 1,733 (1,005–2,837) | 804 (457–1,357) | 830 (511–1,383) | 1,309 (866–1,958) |
|  | **Hemoglobinopathy Total** | **1,344 (463–3,818)** | **517 (236–1,166)** | **4,237 (2,167–8,192)** | **2,567 (1,217–5,558)** | **3,996 (2,093–8,081)** | **8,643 (4,588–17,423)** |
| **Total** | **All ConGDs Total** | **165,812 (104,724–263,283)** | **54,230 (36,952–81,050)** | **136,722 (90,684–207,484)** | **55,797 (36,437–85,940)** | **28,508 (18,929–43,621)** | **38,872 (25,447–60,800)** |
| UI, uncertainty interval. Deaths shown as Number (95% UI). | | | | | | | |

Table S5. Frontier Model Diagnostics (2023).

| **Parameter** | ***τ* = 0.05** | ***τ* = 0.10** |
| --- | --- | --- |
| Pseudo-*R²* | 0.32 | 0.37 |
| Empirical coverage (%) | 4.9% | 8.8% |
| Interior knots (SDI) | 0.608, 0.76 | 0.608, 0.76 |
| Boundary knots (SDI) | [0.203, 0.946] | [0.203, 0.946] |

Table S6. Efficiency Gap Rankings (2023). Panel A. Top-10 (largest gaps).

| **Rank** | **Country** | **SDI Quintile** | **SDI** | **Observed ASMR** | **Frontier ASMR** | **Efficiency Gap** | **Gap %** |
| --- | --- | --- | --- | --- | --- | --- | --- |
| 1 | Azerbaijan | High-middle | 0.705 | 162.44 | 22.78 | 139.66 | 86.0% |
| 2 | Afghanistan | Low | 0.334 | 204.53 | 69.46 | 135.07 | 66.0% |
| 3 | Libya | High-middle | 0.770 | 143.51 | 17.78 | 125.73 | 87.6% |
| 4 | Haiti | Low-middle | 0.464 | 161.20 | 50.53 | 110.68 | 68.7% |
| 5 | Tunisia | Middle | 0.684 | 131.54 | 24.73 | 106.82 | 81.2% |
| 6 | Egypt | Middle | 0.677 | 121.76 | 25.41 | 96.35 | 79.1% |
| 7 | Bolivia (Plurinational State of) | Middle | 0.632 | 120.27 | 29.97 | 90.30 | 75.1% |
| 8 | Lao People's Democratic Republic | Low-middle | 0.515 | 128.52 | 43.79 | 84.73 | 65.9% |
| 9 | Ecuador | Middle | 0.691 | 107.11 | 24.05 | 83.06 | 77.5% |
| 10 | Jordan | High-middle | 0.720 | 101.06 | 21.50 | 79.56 | 78.7% |

Table S6 (continued). Panel B. Bottom-10 (nearest frontier).

| **Rank** | **Country** | **SDI Quintile** | **SDI** | **Observed ASMR** | **Frontier ASMR** | **Efficiency Gap** | **Gap %** |
| --- | --- | --- | --- | --- | --- | --- | --- |
| 1 | Vanuatu | Low-middle | 0.477 | 27.22 | 48.81 | −21.59 | −79.3% |
| 2 | Solomon Islands | Low-middle | 0.458 | 43.74 | 51.31 | −7.57 | −17.3% |
| 3 | Nepal | Low-middle | 0.471 | 42.66 | 49.56 | −6.89 | −16.2% |
| 4 | Bosnia and Herzegovina | High-middle | 0.713 | 16.86 | 22.12 | −5.26 | −31.2% |
| 5 | China | High-middle | 0.728 | 16.15 | 20.92 | −4.77 | −29.6% |
| 6 | Czechia | High | 0.831 | 11.42 | 14.22 | −2.80 | −24.5% |
| 7 | Republic of Korea | High | 0.892 | 9.70 | 11.44 | −1.74 | −17.9% |
| 8 | Cuba | Middle | 0.695 | 23.67 | 23.70 | −0.03 | −0.1% |
| 9 | Micronesia (Federated States of) | Low-middle | 0.597 | 33.87 | 33.87 | −0.00 | −0.0% |
| 10 | Montenegro | High-middle | 0.789 | 16.62 | 16.62 | −0.00 | −0.0% |

Global: 251,423 avoidable deaths (52.4% of total).

Table S7. Sex-stratified ASMR for ConGDs (2023).

| **Condition** | **Male ASMR (95% UI)** | **Female ASMR (95% UI)** | **M:F Ratio** |
| --- | --- | --- | --- |
| **All ConGDs (composite)** | **75.93 (41.65–133.38)** | **73.27 (42.32–123.07)** | **1.04** |
| Congenital heart anomalies | 36.74 (24.77–54.46) | 36.92 (23.86–52.12) | 1.00 |
| Neural tube defects | 6.42 (2.83–12.83) | 5.53 (2.32–11.21) | 1.16 |
| Down syndrome | 3.39 (1.58–6.15) | 2.43 (1.15–4.87) | 1.39 |
| Other chromosomal abnormalities | 2.38 (1.45–4.09) | 3.71 (2.14–6.75) | 0.64 |
| Orofacial clefts | 0.55 (0.07–1.70) | 0.47 (0.08–2.01) | 1.16 |
| Digestive congenital anomalies | 10.46 (5.64–17.43) | 9.40 (5.30–15.90) | 1.11 |
| Urogenital congenital anomalies | 2.39 (1.04–4.80) | 1.44 (0.47–4.68) | 1.66 |
| Congenital musculoskeletal and limb anomalies | 0.99 (0.54–1.62) | 1.42 (0.73–2.74) | 0.70 |
| Other congenital birth defects | 9.42 (2.34–24.31) | 8.50 (4.82–12.90) | 1.11 |
| Sickle cell disorders | 2.01 (0.81–3.95) | 2.65 (1.01–8.40) | 0.76 |
| Thalassemias | 0.99 (0.49–1.69) | 0.68 (0.36–1.31) | 1.45 |
| G6PD deficiency | 0.11 (0.05–0.22) | 0.04 (0.02–0.07) | 2.59 |
| Other hemoglobinopathies and hemolytic anemias | 0.08 (0.05–0.13) | 0.07 (0.05–0.09) | 1.20 |
| G6PD deficiency is X-linked; M:F ratio >1 = male excess. | | | |

Table S8. Regional ConGD mortality burden (1990–2023).

|  | **1990** | | **2023** | | **1990–2023** | |
| --- | --- | --- | --- | --- | --- | --- |
| **GBD Region** | **ASMR (95% UI)** | **PMR (%)** | **ASMR (95% UI)** | **PMR (%)** | **ConGD ASMR Change (%)** | **CMNN ASMR Change (%)** |
| Andean Latin America | 100.79 (66.31–151.44) | 6.43 | 91.09 (67.71–119.08) | 19.24 | -9.6 | -77.8 |
| Caribbean | 124.15 (84.94–182.32) | 8.47 | 96.78 (62.39–148.15) | 13.17 | -22.0 | -54.2 |
| Central Asia | 76.38 (63.22–91.42) | 6.56 | 77.56 (62.90–93.43) | 14.38 | 1.5 | -59.3 |
| Central Europe | 86.86 (57.90–101.14) | 20.33 | 27.22 (22.78–37.69) | 30.96 | -68.7 | -82.4 |
| Central Latin America | 91.93 (77.62–108.81) | 10.10 | 74.62 (61.76–85.10) | 22.52 | -18.8 | -71.3 |
| Central Sub-Saharan Africa | 122.39 (47.72–252.47) | 3.22 | 89.92 (40.26–189.85) | 6.39 | -26.5 | -65.6 |
| East Asia | 121.79 (76.60–185.84) | 11.42 | 16.97 (12.16–23.44) | 20.45 | -86.1 | -95.4 |
| Eastern Europe | 83.23 (56.86–101.69) | 21.81 | 34.82 (26.77–52.45) | 28.25 | -58.2 | -73.7 |
| Eastern Sub-Saharan Africa | 126.36 (58.94–240.90) | 3.08 | 94.23 (47.15–177.69) | 7.98 | -25.4 | -73.9 |
| High-income Asia Pacific | 47.27 (36.40–57.59) | 26.69 | 16.78 (13.19–25.31) | 37.28 | -64.5 | -80.4 |
| High-income North America | 49.64 (37.51–58.35) | 21.93 | 29.29 (22.93–42.25) | 22.97 | -41.0 | -44.2 |
| North Africa and Middle East | 183.17 (112.00–284.50) | 11.77 | 108.24 (72.27–167.14) | 23.04 | -40.9 | -77.1 |
| Oceania | 89.92 (44.33–169.65) | 7.56 | 85.25 (44.82–154.17) | 12.15 | -5.2 | -44.5 |
| South Asia | 98.87 (55.99–166.99) | 3.56 | 78.43 (52.19–120.09) | 11.07 | -20.7 | -77.3 |
| Southeast Asia | 89.21 (52.00–145.94) | 6.20 | 74.68 (46.40–118.19) | 15.53 | -16.3 | -71.9 |
| Southern Latin America | 84.91 (57.62–105.41) | 16.07 | 54.42 (43.02–68.87) | 33.63 | -35.9 | -74.5 |
| Southern Sub-Saharan Africa | 53.80 (29.44–90.27) | 4.07 | 78.10 (48.51–120.40) | 9.29 | 45.2 | -42.2 |
| Tropical Latin America | 79.18 (67.00–92.40) | 8.14 | 65.69 (55.50–84.68) | 24.25 | -17.0 | -79.1 |
| Western Europe | 49.04 (34.24–56.43) | 26.44 | 21.45 (17.08–33.79) | 30.45 | -56.3 | -59.1 |
| Western Sub-Saharan Africa | 119.95 (54.79–231.49) | 2.54 | 104.02 (51.76–196.12) | 5.96 | -13.3 | -65.6 |
| ASMR per 100,000; PMR = proportional mortality ratio; CMNN = communicable, maternal, neonatal, nutritional. | | | | | | |

Table S9. Country-level congenital and genetic disorder mortality burden in children under 5 years across 204 countries, 2023.

| **Country** | **ASMR (95% UI)** | **Deaths (N)** | **PMR (%)** |
| --- | --- | --- | --- |
| Afghanistan | 204.53 (107.47–375.66) | 12,707 | 18.00 |
| Azerbaijan | 162.44 (96.37–256.17) | 1,026 | 16.84 |
| Haiti | 161.20 (86.45–274.89) | 2,654 | 13.03 |
| Libya | 143.51 (93.61–217.68) | 830 | 23.55 |
| Sierra Leone | 138.82 (67.70–255.77) | 1,522 | 5.99 |
| Tunisia | 131.54 (83.21–199.07) | 1,077 | 23.88 |
| Lao People’s Democratic Republic | 128.52 (71.10–218.17) | 1,033 | 13.25 |
| Benin | 127.27 (47.30–303.37) | 2,991 | 8.92 |
| Ethiopia | 124.50 (64.19–222.81) | 20,421 | 10.63 |
| Egypt | 121.76 (71.21–210.69) | 15,208 | 29.65 |
| Bolivia (Plurinational State of) | 120.27 (69.32–196.14) | 1,374 | 20.47 |
| Yemen | 118.22 (68.81–205.12) | 5,870 | 17.19 |
| Myanmar | 118.19 (66.03–203.87) | 6,321 | 13.53 |
| South Sudan | 115.84 (54.82–227.26) | 1,618 | 4.99 |
| Guinea | 115.43 (50.75–223.33) | 2,704 | 6.61 |
| Liberia | 110.15 (48.39–231.45) | 842 | 5.87 |
| Nigeria | 109.54 (49.43–225.58) | 42,483 | 5.92 |
| Sudan | 109.23 (62.59–188.09) | 4,571 | 20.10 |
| Burkina Faso | 108.32 (44.60–221.90) | 4,799 | 8.81 |
| Mali | 108.23 (52.91–202.44) | 4,979 | 5.60 |
| Ecuador | 107.11 (79.24–132.16) | 1,593 | 18.92 |
| Togo | 106.57 (36.82–196.93) | 1,217 | 8.29 |
| Iraq | 105.87 (61.70–171.05) | 4,957 | 24.53 |
| Central African Republic | 105.05 (43.58–236.80) | 1,272 | 4.28 |
| Algeria | 104.50 (62.59–165.87) | 4,663 | 29.11 |
| Zambia | 103.18 (52.32–196.53) | 3,332 | 9.78 |
| Mozambique | 102.70 (51.57–195.48) | 5,747 | 7.79 |
| Guinea-Bissau | 102.36 (42.27–237.59) | 342 | 6.54 |
| Jordan | 101.06 (57.33–155.38) | 1,157 | 38.91 |
| Côte d’Ivoire | 100.90 (48.57–191.20) | 5,015 | 6.53 |
| Eritrea | 100.29 (50.61–190.86) | 986 | 8.32 |
| Zimbabwe | 100.28 (52.15–181.27) | 2,204 | 8.79 |
| Equatorial Guinea | 99.54 (40.97–195.65) | 203 | 9.59 |
| Turkmenistan | 98.50 (63.98–132.45) | 588 | 17.04 |
| Fiji | 97.95 (59.38–149.20) | 87 | 21.94 |
| Rwanda | 96.29 (40.89–191.74) | 1,704 | 8.90 |
| Angola | 95.67 (45.59–179.08) | 5,651 | 7.96 |
| Djibouti | 95.59 (42.87–183.88) | 163 | 9.68 |
| Paraguay | 95.18 (65.36–133.74) | 516 | 26.68 |
| Burundi | 94.09 (39.04–199.97) | 2,190 | 5.48 |
| Ghana | 93.35 (43.58–168.21) | 4,006 | 10.25 |
| Bangladesh | 93.08 (52.04–159.20) | 15,056 | 14.62 |
| Niger | 92.23 (36.98–183.10) | 5,020 | 3.51 |
| Timor-Leste | 91.74 (53.27–158.10) | 174 | 12.19 |
| Somalia | 91.42 (42.55–186.00) | 3,763 | 4.98 |
| Guyana | 91.35 (65.16–119.65) | 68 | 16.02 |
| Papua New Guinea | 91.22 (46.55–169.00) | 1,500 | 11.71 |
| Malawi | 91.11 (41.34–184.08) | 2,665 | 7.86 |
| Comoros | 90.23 (42.24–175.37) | 96 | 9.42 |
| Panama | 89.13 (64.67–109.07) | 309 | 31.07 |
| Pakistan | 88.72 (50.52–141.18) | 27,350 | 8.49 |
| Suriname | 88.64 (57.40–134.17) | 40 | 15.34 |
| Morocco | 87.96 (51.82–143.13) | 2,304 | 25.88 |
| Democratic Republic of the Congo | 87.18 (35.86–196.46) | 12,733 | 6.05 |
| Chad | 86.59 (39.74–167.41) | 3,461 | 3.67 |
| Mexico | 86.35 (68.68–104.23) | 7,937 | 26.35 |
| Cameroon | 86.02 (40.43–160.94) | 4,418 | 5.94 |
| Madagascar | 85.38 (39.17–171.67) | 3,952 | 6.75 |
| Uganda | 85.12 (35.81–171.16) | 6,390 | 7.42 |
| Gambia | 84.50 (33.28–205.93) | 307 | 7.24 |
| Senegal | 83.14 (37.74–161.66) | 2,252 | 9.28 |
| Venezuela (Bolivarian Republic of) | 82.77 (59.80–110.57) | 1,288 | 13.34 |
| Indonesia | 80.81 (45.63–139.23) | 17,619 | 15.16 |
| Seychelles | 79.00 (47.46–122.54) | 6 | 31.64 |
| Uzbekistan | 78.94 (48.25–97.45) | 3,254 | 11.39 |
| Türkiye | 77.67 (47.45–117.93) | 4,209 | 23.00 |
| Bhutan | 77.36 (43.41–129.30) | 39 | 13.59 |
| United Republic of Tanzania | 76.19 (34.87–152.74) | 7,523 | 8.36 |
| Sao Tome and Principe | 76.06 (34.97–137.59) | 17 | 11.04 |
| Congo | 75.86 (33.88–151.19) | 584 | 7.62 |
| Philippines | 75.62 (51.41–109.04) | 7,612 | 14.25 |
| Gabon | 75.23 (31.29–148.62) | 178 | 10.24 |
| Eswatini | 74.82 (40.04–131.30) | 100 | 7.30 |
| India | 74.32 (48.08–112.56) | 81,780 | 11.83 |
| El Salvador | 74.11 (48.88–106.70) | 326 | 26.04 |
| Peru | 72.46 (48.36–100.59) | 2,223 | 18.78 |
| South Africa | 71.88 (46.34–104.09) | 3,756 | 10.07 |
| Kyrgyzstan | 71.53 (56.53–91.41) | 567 | 17.84 |
| Cambodia | 69.76 (37.96–124.87) | 1,212 | 15.46 |
| Lebanon | 69.38 (42.49–107.20) | 293 | 17.91 |
| Kazakhstan | 67.98 (53.48–98.30) | 1,410 | 25.45 |
| Nicaragua | 67.50 (44.96–94.94) | 428 | 24.99 |
| Mauritius | 67.30 (44.15–89.22) | 43 | 23.36 |
| Cabo Verde | 67.25 (34.01–117.04) | 25 | 14.31 |
| Brunei Darussalam | 66.77 (42.82–101.09) | 19 | 36.06 |
| Mauritania | 65.59 (29.36–121.21) | 437 | 7.65 |
| Iran (Islamic Republic of) | 65.40 (36.52–104.10) | 3,657 | 30.52 |
| Guatemala | 64.86 (43.14–84.45) | 1,105 | 13.79 |
| Brazil | 64.61 (53.97–84.30) | 9,601 | 24.13 |
| Palestine | 64.07 (41.45–95.64) | 420 | 12.54 |
| Lesotho | 63.64 (31.02–117.87) | 129 | 4.89 |
| Maldives | 63.09 (38.08–97.03) | 20 | 21.85 |
| Nauru | 62.90 (35.59–104.98) | 1 | 11.82 |
| Kuwait | 61.99 (49.15–80.58) | 155 | 39.64 |
| Tajikistan | 61.92 (38.27–101.02) | 835 | 11.92 |
| Kiribati | 61.30 (32.31–111.49) | 9 | 7.96 |
| Trinidad and Tobago | 60.80 (45.64–78.93) | 44 | 27.34 |
| Republic of Moldova | 58.59 (39.51–79.00) | 86 | 27.46 |
| Colombia | 57.14 (44.94–66.54) | 2,121 | 24.75 |
| Costa Rica | 57.00 (45.97–72.23) | 164 | 38.51 |
| Namibia | 56.93 (29.98–99.63) | 194 | 7.54 |
| Argentina | 56.10 (42.98–70.63) | 1,561 | 32.29 |
| Botswana | 55.38 (29.36–98.39) | 139 | 11.29 |
| Palau | 54.37 (30.33–92.47) | 1 | 11.85 |
| Jamaica | 53.84 (37.84–71.02) | 101 | 17.15 |
| Tuvalu | 52.60 (29.16–92.09) | 1 | 11.58 |
| Armenia | 52.59 (37.40–69.84) | 90 | 22.71 |
| Viet Nam | 52.33 (29.48–85.85) | 4,108 | 23.15 |
| Oman | 51.97 (28.06–90.24) | 225 | 33.88 |
| Chile | 51.85 (40.37–69.87) | 502 | 38.29 |
| Dominican Republic | 51.77 (30.49–80.20) | 526 | 10.84 |
| Guam | 51.75 (37.84–73.64) | 6 | 17.02 |
| Saudi Arabia | 51.29 (30.45–78.97) | 1,302 | 27.44 |
| Belize | 48.37 (34.64–65.68) | 17 | 18.41 |
| Democratic People’s Republic of Korea | 48.04 (27.32–80.73) | 716 | 18.27 |
| Honduras | 46.39 (25.85–78.10) | 561 | 22.54 |
| Mongolia | 46.37 (28.22–70.29) | 177 | 15.42 |
| Ukraine | 45.91 (32.88–66.04) | 580 | 30.19 |
| Bahrain | 45.88 (25.70–72.22) | 40 | 37.52 |
| Barbados | 45.40 (33.74–59.05) | 6 | 24.05 |
| Northern Mariana Islands | 44.81 (23.25–77.79) | 1 | 18.86 |
| Greenland | 44.55 (25.83–72.17) | 2 | 18.01 |
| Albania | 44.37 (28.94–66.98) | 53 | 26.18 |
| Bahamas | 44.22 (31.90–64.87) | 9 | 20.44 |
| Tonga | 44.18 (24.82–76.29) | 5 | 20.22 |
| Solomon Islands | 43.74 (23.03–78.12) | 49 | 22.62 |
| Sri Lanka | 43.41 (29.39–62.00) | 617 | 27.60 |
| Kenya | 43.13 (19.76–85.90) | 2,563 | 6.22 |
| Grenada | 42.97 (29.72–66.70) | 3 | 14.47 |
| Nepal | 42.66 (24.24–73.71) | 1,210 | 7.23 |
| Uruguay | 42.03 (30.81–60.98) | 73 | 35.50 |
| Saint Kitts and Nevis | 41.57 (31.45–56.32) | 1 | 17.84 |
| Syrian Arab Republic | 41.30 (27.60–63.14) | 546 | 20.53 |
| Saint Vincent and the Grenadines | 40.89 (27.61–65.56) | 3 | 18.91 |
| Samoa | 40.13 (22.05–69.77) | 12 | 12.46 |
| Malaysia | 38.44 (24.30–57.07) | 885 | 27.32 |
| Marshall Islands | 37.67 (20.84–67.21) | 2 | 11.21 |
| Bulgaria | 37.07 (28.78–51.21) | 112 | 32.44 |
| Saint Lucia | 36.39 (25.53–55.33) | 3 | 17.96 |
| Niue | 36.26 (19.95–62.54) | 0 | 16.95 |
| Dominica | 36.20 (22.83–55.27) | 1 | 20.76 |
| Malta | 35.71 (26.99–46.63) | 8 | 41.04 |
| Romania | 35.45 (27.94–50.57) | 323 | 28.73 |
| Antigua and Barbuda | 34.66 (23.53–59.90) | 2 | 21.01 |
| Qatar | 34.65 (20.16–53.87) | 53 | 33.76 |
| Georgia | 33.98 (24.71–47.29) | 76 | 18.98 |
| Belarus | 33.91 (25.37–48.85) | 148 | 40.28 |
| Micronesia (Federated States of) | 33.87 (21.10–54.33) | 3 | 13.36 |
| Puerto Rico | 33.32 (23.90–53.35) | 28 | 21.24 |
| Thailand | 33.22 (20.90–48.60) | 879 | 22.09 |
| Russian Federation | 32.76 (24.49–51.45) | 2,049 | 27.12 |
| American Samoa | 31.92 (16.03–54.73) | 1 | 14.84 |
| Bermuda | 30.10 (21.84–41.01) | 1 | 31.99 |
| United States of America | 29.61 (22.98–42.85) | 5,486 | 22.65 |
| Poland | 29.54 (23.21–42.91) | 523 | 40.92 |
| United States Virgin Islands | 28.09 (18.19–52.42) | 1 | 21.12 |
| United Arab Emirates | 28.02 (16.02–45.36) | 122 | 30.36 |
| Tokelau | 27.96 (15.52–47.45) | 0 | 14.39 |
| Slovakia | 27.38 (17.46–39.96) | 76 | 27.20 |
| Vanuatu | 27.22 (14.19–50.29) | 12 | 8.50 |
| Ireland | 26.26 (19.95–39.38) | 78 | 42.44 |
| Greece | 26.12 (19.85–38.74) | 107 | 33.71 |
| Canada | 26.10 (20.42–37.99) | 484 | 27.33 |
| Switzerland | 25.72 (19.70–37.11) | 112 | 38.37 |
| Denmark | 25.64 (18.83–39.25) | 78 | 36.15 |
| United Kingdom | 24.87 (19.94–39.23) | 886 | 28.59 |
| Croatia | 24.47 (18.62–37.41) | 43 | 31.60 |
| New Zealand | 24.26 (18.54–37.66) | 72 | 28.21 |
| Netherlands | 24.21 (18.08–39.00) | 210 | 31.95 |
| Hungary | 23.81 (16.45–42.78) | 108 | 30.34 |
| Cuba | 23.67 (14.10–45.40) | 122 | 22.42 |
| Germany | 23.32 (18.30–34.74) | 921 | 33.10 |
| Lithuania | 23.10 (17.22–37.30) | 29 | 36.86 |
| North Macedonia | 23.05 (16.86–31.27) | 22 | 21.82 |
| Taiwan | 22.94 (16.53–37.27) | 176 | 28.26 |
| Israel | 22.85 (17.06–36.84) | 215 | 36.01 |
| Iceland | 22.48 (16.71–34.45) | 5 | 33.99 |
| Andorra | 21.69 (12.77–32.83) | 1 | 32.90 |
| Australia | 21.61 (16.36–32.93) | 325 | 29.87 |
| San Marino | 21.41 (11.68–35.83) | 0 | 28.79 |
| Cook Islands | 21.06 (10.89–37.54) | 0 | 13.97 |
| Portugal | 20.72 (14.68–35.06) | 87 | 31.08 |
| Serbia | 20.64 (14.18–29.50) | 71 | 20.74 |
| Latvia | 19.83 (13.69–34.53) | 18 | 29.54 |
| Austria | 19.64 (14.51–31.47) | 84 | 33.64 |
| France | 19.23 (13.68–33.73) | 696 | 24.41 |
| Finland | 19.07 (13.31–32.20) | 45 | 41.14 |
| Japan | 18.82 (14.14–29.89) | 778 | 43.08 |
| Spain | 18.75 (13.26–33.37) | 332 | 29.23 |
| Norway | 17.87 (12.10–35.54) | 49 | 40.05 |
| Italy | 17.77 (13.25–27.36) | 371 | 32.12 |
| Sweden | 17.11 (12.29–29.52) | 96 | 35.52 |
| Bosnia and Herzegovina | 16.86 (11.54–24.49) | 24 | 17.05 |
| Cyprus | 16.78 (8.95–28.15) | 13 | 23.17 |
| Montenegro | 16.62 (10.77–24.07) | 6 | 18.43 |
| Monaco | 16.57 (8.57–29.47) | 0 | 22.78 |
| China | 16.15 (11.25–22.54) | 10,037 | 20.53 |
| Singapore | 16.11 (11.31–28.85) | 41 | 45.62 |
| Belgium | 14.79 (10.82–24.41) | 87 | 28.79 |
| Slovenia | 14.04 (10.04–24.66) | 13 | 32.27 |
| Estonia | 13.05 (8.37–25.74) | 9 | 26.75 |
| Luxembourg | 12.69 (8.66–25.88) | 4 | 23.13 |
| Czechia | 11.42 (7.45–23.97) | 64 | 24.03 |
| Republic of Korea | 9.70 (6.32–13.97) | 132 | 20.22 |
| ASMR, age-standardized mortality rate per 100,000; PMR, proportional mortality ratio; UI, uncertainty interval. Countries ranked by ASMR (descending). | | | |

Table S10. Sensitivity of avoidable ConGD deaths estimates to frontier specification.

| **SDI Quintile** | **Countries (n)** | **Total deaths** | **Main frontier: avoidable (n)** | **Main: %** | **HQ-VR frontier: avoidable (n)** | **HQ-VR: %** |
| --- | --- | --- | --- | --- | --- | --- |
| **Global** | **204** | **479,556** | **251,423** | **52.4%** | **248,559** | **51.8%** |
| High | 48 | 16,032 | 8,490 | 53.0% | 8,140 | 50.8% |
| High-middle | 49 | 33,030 | 15,568 | 47.1% | 12,135 | 36.7% |
| Middle | 39 | 90,829 | 61,561 | 67.8% | 46,672 | 51.4% |
| Low-middle | 42 | 223,618 | 120,337 | 53.8% | 110,466 | 49.4% |
| Low | 26 | 116,047 | 45,467 | 39.2% | 71,146 | 61.3% |
| Main frontier: *τ* = 0.05 quantile regression on log-ASMR vs SDI (natural cubic spline, *df* = 3), all 204 countries. HQ-VR frontier: same specification but fitted only on 71 countries with GBD data quality rating ≥ 4 stars, then applied to all countries to recompute efficiency gaps. Source of star ratings: the GBD 2019 data-quality appendix. | | | | | | |

Table S11. Sensitivity analysis excluding high-consanguinity (MENA + Pakistan + Afghanistan) countries from the frontier.

| **Specification** | **Countries in frontier fit** | **Avoidable deaths (n)** | **Avoidable share (%)** | **Scope** |
| --- | --- | --- | --- | --- |
| Main (all countries) | 204 | 251,423 | 52.4% | Global, 204 countries |
| MENA excluded from frontier fit, gaps reported for non-MENA only | 182 | 192,470 | 49.6% | Non-MENA, 182 countries |
| The MENA region has documented consanguinity rates of 20–50% (Bittles & Black, PNAS 2010), elevating baseline autosomal recessive ConGD incidence independently of health system performance. MENA countries (n=22 in data): Afghanistan, Algeria, Bahrain, Egypt, Iran (Islamic Republic of), Iraq, Jordan, Kuwait, Lebanon, Libya, Morocco, Oman, Pakistan, Palestine, Qatar, Saudi Arabia, Sudan, Syrian Arab Republic, Tunisia, Türkiye, United Arab Emirates, Yemen. | | | | |

Table S12. Estimated annual percentage change (EAPC) of ConGD age-standardized mortality rate, 1990–2023.

| **Location** | **EAPC (%)** | **95% CI (%)** | **Trend** |
| --- | --- | --- | --- |
| Global | −1.35 | −1.45, −1.26 | Decreasing |
| Andean Latin America | −0.37 | −0.55, −0.19 | Decreasing |
| Caribbean | −0.68 | −0.77, −0.59 | Decreasing |
| Central Asia | 0.40 | 0.18, 0.61 | Increasing |
| Central Europe | −3.58 | −3.68, −3.48 | Decreasing |
| Central Europe, Eastern Europe, and Central Asia | −1.64 | −1.81, −1.48 | Decreasing |
| Central Latin America | −0.59 | −0.68, −0.49 | Decreasing |
| Central Sub-Saharan Africa | −1.06 | −1.18, −0.94 | Decreasing |
| East Asia | −6.74 | −7.60, −5.86 | Decreasing |
| Eastern Europe | −3.15 | −3.55, −2.75 | Decreasing |
| Eastern Sub-Saharan Africa | −0.91 | −1.10, −0.72 | Decreasing |
| High-income | −2.06 | −2.16, −1.96 | Decreasing |
| High-income Asia Pacific | −3.49 | −3.66, −3.32 | Decreasing |
| High-income North America | −1.48 | −1.61, −1.36 | Decreasing |
| Latin America and Caribbean | −0.59 | −0.70, −0.49 | Decreasing |
| North Africa and Middle East | −1.94 | −2.08, −1.79 | Decreasing |
| Oceania | −0.32 | −0.52, −0.12 | Decreasing |
| South Asia | −0.84 | −1.03, −0.66 | Decreasing |
| Southeast Asia | −0.89 | −1.02, −0.76 | Decreasing |
| Southeast Asia, East Asia, and Oceania | −3.70 | −4.06, −3.33 | Decreasing |
| Southern Latin America | −1.43 | −1.67, −1.19 | Decreasing |
| Southern Sub-Saharan Africa | 1.54 | 1.37, 1.72 | Increasing |
| Sub-Saharan Africa | −0.61 | −0.75, −0.47 | Decreasing |
| Tropical Latin America | −0.69 | −0.94, −0.44 | Decreasing |
| Western Europe | −2.59 | −2.78, −2.40 | Decreasing |
| Western Sub-Saharan Africa | −0.52 | −0.64, −0.40 | Decreasing |
| EAPC derived from a log-linear regression of ln(ASMR) on calendar year; EAPC = 100 × [exp(β) − 1] with 95% CI from the standard error of β. Trend: Decreasing (upper CI < 0), Increasing (lower CI > 0), or Stable (CI spans 0). | | | |

Table S13. Joinpoint regression of ConGD age-standardized mortality rate, 1990–2023. Inflection years detected via segmented regression on ln(ASMR); model order selected by AICc (up to 2 joinpoints).

| **Location** | **Joinpoints** | **Segments** | **AAPC** | **AAPC 95% CI** |
| --- | --- | --- | --- | --- |
| Global | 1996, 2018 | 1990–1996: −0.44% (95% CI −0.72, −0.16) \| 1996–2018: −1.67% (95% CI −1.73, −1.62) \| 2018–2023: 1.08% (95% CI 0.73, 1.44) | −1.03 | −1.17, −0.89 |
| Andean Latin America | 2004, 2018 | 1990–2004: −1.40% (95% CI −1.79, −1.00) \| 2004–2018: 0.66% (95% CI 0.16, 1.16) \| 2018–2023: −2.03% (95% CI −3.58, −0.45) | −0.62 | −1.23, −0.00 |
| Caribbean | 2004, 2007 | 1990–2004: −1.06% (95% CI −1.31, −0.80) \| 2004–2007: 1.01% (95% CI −2.05, 4.16) \| 2007–2023: −0.83% (95% CI −1.06, −0.59) | −0.76 | −1.26, −0.25 |
| Central Asia | 1997, 2005 | 1990–1997: −2.99% (95% CI −4.35, −1.61) \| 1997–2005: 2.86% (95% CI 1.68, 4.06) \| 2005–2023: −0.21% (95% CI −0.52, 0.11) | −0.05 | −0.80, 0.70 |
| Central Europe | 2008, 2012 | 1990–2008: −3.43% (95% CI −3.51, −3.36) \| 2008–2012: −6.35% (95% CI −7.57, −5.12) \| 2012–2023: −2.43% (95% CI −2.58, −2.28) | −3.45 | −3.69, −3.21 |
| Central Europe, Eastern Europe, and Central Asia | 2006, 2017 | 1990–2006: −0.88% (95% CI −1.07, −0.69) \| 2006–2017: −3.04% (95% CI −3.33, −2.75) \| 2017–2023: 1.45% (95% CI 0.59, 2.31) | −1.18 | −1.52, −0.83 |
| Central Latin America | 2013 | 1990–2013: −0.29% (95% CI −0.36, −0.22) \| 2013–2023: −1.55% (95% CI −1.75, −1.35) | −0.67 | −0.78, −0.56 |
| Central Sub-Saharan Africa | 2000, 2006 | 1990–2000: −0.63% (95% CI −0.92, −0.35) \| 2000–2006: −3.13% (95% CI −4.04, −2.21) \| 2006–2023: −0.40% (95% CI −0.53, −0.26) | −0.96 | −1.29, −0.64 |
| East Asia | 2004 | 1990–2004: −0.68% (95% CI −1.44, 0.07) \| 2004–2023: −10.75% (95% CI −11.22, −10.27) | −6.48 | −7.07, −5.88 |
| Eastern Europe | 2002, 2019 | 1990–2002: 0.33% (95% CI −0.05, 0.72) \| 2002–2019: −5.11% (95% CI −5.35, −4.87) \| 2019–2023: −0.38% (95% CI −2.63, 1.93) | −2.56 | −3.09, −2.02 |
| Eastern Sub-Saharan Africa | 1995, 2003 | 1990–1995: −0.62% (95% CI −1.18, −0.06) \| 1995–2003: −2.80% (95% CI −3.02, −2.57) \| 2003–2023: −0.02% (95% CI −0.09, 0.05) | −0.79 | −0.97, −0.60 |
| High-income | 1996, 2020 | 1990–1996: −3.46% (95% CI −3.80, −3.12) \| 1996–2020: −2.00% (95% CI −2.05, −1.95) \| 2020–2023: 1.19% (95% CI −0.13, 2.53) | −1.98 | −2.20, −1.75 |
| High-income Asia Pacific | 2005, 2020 | 1990–2005: −4.29% (95% CI −4.49, −4.08) \| 2005–2020: −3.07% (95% CI −3.33, −2.81) \| 2020–2023: 0.39% (95% CI −1.39, 2.20) | −3.31 | −3.68, −2.93 |
| High-income North America | 1995, 2020 | 1990–1995: −4.00% (95% CI −4.64, −3.35) \| 1995–2020: −1.34% (95% CI −1.42, −1.27) \| 2020–2023: 1.04% (95% CI −0.95, 3.07) | −1.53 | −1.87, −1.19 |
| Latin America and Caribbean | 1996, 2013 | 1990–1996: 0.22% (95% CI −0.11, 0.55) \| 1996–2013: −0.37% (95% CI −0.45, −0.28) \| 2013–2023: −1.65% (95% CI −1.84, −1.46) | −0.65 | −0.81, −0.49 |
| North Africa and Middle East | 2004, 2018 | 1990–2004: −1.03% (95% CI −1.17, −0.88) \| 2004–2018: −2.93% (95% CI −3.08, −2.77) \| 2018–2023: 0.61% (95% CI −0.15, 1.38) | −1.58 | −1.83, −1.34 |
| Oceania | 2008, 2018 | 1990–2008: 0.62% (95% CI 0.43, 0.81) \| 2008–2018: −2.42% (95% CI −2.98, −1.86) \| 2018–2023: 1.91% (95% CI 0.83, 3.01) | −0.11 | −0.54, 0.33 |
| South Asia | 2001, 2015 | 1990–2001: −1.66% (95% CI −1.97, −1.35) \| 2001–2015: −1.10% (95% CI −1.30, −0.90) \| 2015–2023: 1.78% (95% CI 1.26, 2.31) | −0.59 | −0.90, −0.27 |
| Southeast Asia | 1993, 2018 | 1990–1993: 0.99% (95% CI −1.31, 3.35) \| 1993–2018: −1.19% (95% CI −1.28, −1.11) \| 2018–2023: 2.28% (95% CI 1.49, 3.08) | −0.47 | −0.87, −0.07 |
| Southeast Asia, East Asia, and Oceania | 2003, 2019 | 1990–2003: −0.94% (95% CI −1.22, −0.66) \| 2003–2019: −5.82% (95% CI −6.04, −5.60) \| 2019–2023: 3.70% (95% CI 1.72, 5.72) | −2.74 | −3.20, −2.28 |
| Southern Latin America | 2013, 2020 | 1990–2013: −0.65% (95% CI −0.76, −0.54) \| 2013–2020: −5.36% (95% CI −6.05, −4.67) \| 2020–2023: 4.73% (95% CI 1.91, 7.62) | −1.16 | −1.64, −0.67 |
| Southern Sub-Saharan Africa | 2003, 2005 | 1990–2003: 0.22% (95% CI −0.31, 0.76) \| 2003–2005: 5.46% (95% CI 0.20, 10.99) \| 2005–2023: 1.68% (95% CI 1.35, 2.02) | 1.34 | 0.63, 2.07 |
| Sub-Saharan Africa | 1996, 2005 | 1990–1996: −0.26% (95% CI −0.64, 0.12) \| 1996–2005: −1.94% (95% CI −2.14, −1.74) \| 2005–2023: 0.10% (95% CI 0.03, 0.16) | −0.52 | −0.69, −0.36 |
| Tropical Latin America | 1997, 2012 | 1990–1997: 1.99% (95% CI 1.61, 2.37) \| 1997–2012: −0.49% (95% CI −0.64, −0.35) \| 2012–2023: −2.64% (95% CI −2.86, −2.41) | −0.68 | −0.90, −0.46 |
| Western Europe | 2002, 2018 | 1990–2002: −3.76% (95% CI −3.96, −3.56) \| 2002–2018: −2.45% (95% CI −2.58, −2.32) \| 2018–2023: 0.74% (95% CI 0.14, 1.33) | −2.44 | −2.67, −2.22 |
| Western Sub-Saharan Africa | 1997, 2005 | 1990–1997: −0.14% (95% CI −0.43, 0.16) \| 1997–2005: −1.85% (95% CI −2.14, −1.56) \| 2005–2023: 0.14% (95% CI 0.05, 0.22) | −0.40 | −0.58, −0.22 |
| APC: annual percentage change within a segment; AAPC: average APC across the full 1990–2023 period, weighted by segment length. Negative values indicate decline. | | | | |

Table S14. Kitagawa decomposition of the 1990–2023 change in ConGD under-5 deaths.

| **Location** | **Deaths 1990** | **Deaths 2023** | **Δ Deaths** | **Population effect** | **ASMR-change effect** |
| --- | --- | --- | --- | --- | --- |
| Global | 649,326 | 479,942 | −169,384 | +25,916 | −195,300 |
| Andean Latin America | 5,395 | 5,189 | −206 | +330 | −536 |
| Caribbean | 5,126 | 3,755 | −1,371 | −276 | −1,096 |
| Central Asia | 7,362 | 8,023 | +661 | +542 | +118 |
| Central Europe | 8,026 | 1,461 | −6,565 | −2,209 | −4,356 |
| Central Europe, Eastern Europe, and Central Asia | 29,853 | 12,402 | −17,451 | −8,139 | −9,312 |
| Central Latin America | 20,922 | 14,238 | −6,685 | −3,065 | −3,620 |
| Central Sub-Saharan Africa | 12,949 | 20,621 | +7,672 | +13,114 | −5,442 |
| East Asia | 139,737 | 10,929 | −128,808 | −34,908 | −93,900 |
| Eastern Europe | 14,465 | 2,919 | −11,546 | −5,310 | −6,235 |
| Eastern Sub-Saharan Africa | 45,219 | 63,166 | +17,948 | +34,461 | −16,514 |
| High-income | 31,902 | 13,965 | −17,937 | −3,398 | −14,539 |
| High-income Asia Pacific | 4,769 | 969 | −3,800 | −1,382 | −2,418 |
| High-income North America | 10,769 | 5,972 | −4,798 | −516 | −4,281 |
| Latin America and Caribbean | 45,312 | 33,299 | −12,013 | −4,749 | −7,264 |
| North Africa and Middle East | 189,817 | 128,847 | −60,969 | +22,452 | −83,421 |
| Oceania | 931 | 1,764 | +833 | +905 | −73 |
| South Asia | 297,770 | 250,869 | −46,901 | +16,560 | −63,461 |
| Southeast Asia | 52,305 | 40,579 | −11,726 | −3,515 | −8,211 |
| Southeast Asia, East Asia, and Oceania | 192,973 | 53,271 | −139,701 | −41,448 | −98,253 |
| Southern Latin America | 4,394 | 2,136 | −2,257 | −870 | −1,387 |
| Southern Sub-Saharan Africa | 4,057 | 6,522 | +2,466 | +535 | +1,931 |
| Sub-Saharan Africa | 105,493 | 177,146 | +71,653 | +98,554 | −26,901 |
| Tropical Latin America | 13,869 | 10,118 | −3,751 | −1,531 | −2,220 |
| Western Europe | 11,278 | 4,491 | −6,787 | −727 | −6,060 |
| Western Sub-Saharan Africa | 43,269 | 86,837 | +43,568 | +53,086 | −9,518 |
| Kitagawa decomposition: ΔDeaths = ΔPopulation × mean(ASMR) + mean(Population) × ΔASMR. Population effect = contribution of under-5 population change; ASMR-change effect = contribution of mortality-rate change. Their sum approximates ΔDeaths (small residual from the interaction term). | | | | | |

Table S15. Disability-adjusted life years (DALYs) attributable to congenital and genetic disorders in children under 5, 1990 vs 2023, with age-standardized rates and estimated annual percentage change.

|  | **DALY count** | | **Age-standardized DALY rate (per 100,000)** | | **Trend** | | |
| --- | --- | --- | --- | --- | --- | --- | --- |
| **Location** | **DALYs 1990 (95% UI)** | **DALYs 2023 (95% UI)** | **ASR 1990 (95% UI)** | **ASR 2023 (95% UI)** | **ASR change (%)** | **EAPC (%)** | **EAPC 95% CI** |
| Global | 60,142,605 (41,546,764–86,485,151) | 44,985,875 (30,119,517–67,984,723) | 9,790.5 (6,763.3–14,078.7) | 6,995.8 (4,683.9–10,572.4) | −28.54 | −1.31 | −1.40, −1.21 |
| High SDI | 13,714,477 (10,507,922–17,767,125) | 3,668,225 (3,070,692–4,690,055) | 8,252.0 (6,322.6–10,690.5) | 3,052.8 (2,555.5–3,903.1) | −63.01 | −3.51 | −3.76, −3.26 |
| High-middle SDI | 12,252,094 (9,162,292–16,800,118) | 4,748,101 (3,781,518–6,118,819) | 9,732.6 (7,278.2–13,345.4) | 4,908.0 (3,908.8–6,324.8) | −49.57 | −2.46 | −2.64, −2.28 |
| Middle SDI | 7,701,308 (5,150,376–11,228,997) | 6,035,294 (4,123,677–8,941,731) | 9,629.4 (6,439.8–14,040.3) | 7,364.7 (5,032.0–10,911.3) | −23.52 | −1.15 | −1.26, −1.03 |
| Low-middle SDI | 10,144,474 (6,266,623–16,095,614) | 8,039,389 (5,442,298–12,571,392) | 9,940.2 (6,140.4–15,771.5) | 7,381.9 (4,997.2–11,543.3) | −25.74 | −0.95 | −1.04, −0.86 |
| Low SDI | 16,281,312 (8,721,497–27,998,426) | 22,458,860 (13,167,996–37,072,692) | 11,656.8 (6,244.3–20,045.8) | 9,565.6 (5,608.5–15,789.8) | −17.94 | −0.72 | −0.88, −0.57 |
| Andean Latin America | 499,238 (330,486–744,811) | 479,558 (358,028–625,422) | 9,326.1 (6,173.7–13,913.7) | 8,417.9 (6,284.6–10,978.3) | −9.74 | −0.37 | −0.54, −0.19 |
| Australasia | 66,655 (48,698–79,504) | 40,631 (31,599–60,100) | 4,311.6 (3,150.1–5,142.7) | 2,256.6 (1,755.0–3,337.9) | −47.66 | −1.88 | −2.02, −1.75 |
| Caribbean | 471,312 (324,724–688,156) | 348,623 (228,232–528,247) | 11,414.8 (7,864.5–16,666.6) | 8,986.3 (5,883.0–13,616.4) | −21.27 | −0.65 | −0.74, −0.57 |
| Central Asia | 692,357 (572,633–829,207) | 753,308 (611,728–905,900) | 7,183.1 (5,941.0–8,602.9) | 7,283.0 (5,914.2–8,758.3) | 1.39 | 0.37 | 0.16, 0.58 |
| Central Europe | 744,264 (503,097–867,152) | 144,485 (120,822–196,462) | 8,055.1 (5,444.9–9,385.1) | 2,692.0 (2,251.1–3,660.4) | −66.58 | −3.39 | −3.49, −3.29 |
| Central Latin America | 1,961,834 (1,654,694–2,331,702) | 1,341,350 (1,107,983–1,532,182) | 8,620.0 (7,270.4–10,245.1) | 7,030.4 (5,807.3–8,030.7) | −18.44 | −0.58 | −0.67, −0.49 |
| Central Sub-Saharan Africa | 1,202,889 (491,707–2,434,099) | 1,932,827 (896,551–3,991,356) | 11,369.4 (4,647.5–23,006.4) | 8,427.8 (3,909.3–17,403.8) | −25.87 | −1.03 | −1.14, −0.91 |
| East Asia | 12,812,909 (8,112,075–19,456,781) | 1,124,776 (823,354–1,535,363) | 11,167.3 (7,070.2–16,957.9) | 1,746.0 (1,278.1–2,383.4) | −84.36 | −6.38 | −7.19, −5.57 |
| Eastern Europe | 1,350,130 (937,379–1,648,443) | 286,668 (222,375–423,165) | 7,768.5 (5,393.6–9,484.9) | 3,419.9 (2,652.9–5,048.3) | −55.98 | −2.98 | −3.36, −2.60 |
| Eastern Sub-Saharan Africa | 4,181,067 (2,000,068–7,867,437) | 5,879,656 (3,021,829–10,914,176) | 11,683.4 (5,588.9–21,984.3) | 8,771.5 (4,508.1–16,282.3) | −24.92 | −0.89 | −1.08, −0.71 |
| High-income Asia Pacific | 469,776 (360,130–575,489) | 109,516 (85,379–161,627) | 4,656.4 (3,569.6–5,704.3) | 1,896.8 (1,478.8–2,799.4) | −59.26 | −3.03 | −3.19, −2.87 |
| High-income North America | 1,016,464 (776,067–1,189,722) | 578,776 (457,769–821,080) | 4,685.1 (3,577.1–5,483.7) | 2,838.9 (2,245.3–4,027.3) | −39.41 | −1.39 | −1.51, −1.27 |
| North Africa and Middle East | 8,670,306 (5,355,426–13,385,263) | 5,948,717 (4,019,457–9,117,667) | 16,733.2 (10,335.7–25,832.9) | 9,994.6 (6,753.2–15,318.8) | −40.27 | −1.90 | −2.04, −1.76 |
| Oceania | 86,227 (43,227–160,830) | 164,127 (88,430–293,485) | 8,329.7 (4,175.9–15,536.6) | 7,933.6 (4,274.5–14,186.5) | −4.76 | −0.30 | −0.49, −0.11 |
| South Asia | 13,879,807 (8,010,726–23,148,273) | 11,753,192 (7,926,420–17,800,677) | 9,217.2 (5,319.7–15,372.2) | 7,349.2 (4,956.3–11,130.7) | −20.27 | −0.83 | −1.00, −0.65 |
| Southeast Asia | 4,856,361 (2,877,542–7,859,828) | 3,780,959 (2,385,492–5,917,209) | 8,283.2 (4,908.0–13,406.0) | 6,958.0 (4,390.0–10,889.3) | −16.00 | −0.87 | −1.00, −0.74 |
| Southern Latin America | 411,533 (282,071–509,907) | 205,076 (162,851–258,350) | 7,952.9 (5,451.0–9,853.9) | 5,224.6 (4,148.9–6,581.9) | −34.30 | −1.35 | −1.58, −1.12 |
| Southern Sub-Saharan Africa | 383,052 (215,064–631,055) | 608,344 (385,208–928,738) | 5,080.1 (2,852.2–8,369.1) | 7,284.2 (4,612.4–11,120.5) | 43.39 | 1.49 | 1.32, 1.66 |
| Tropical Latin America | 1,289,894 (1,091,674–1,503,878) | 944,964 (798,874–1,212,150) | 7,364.2 (6,232.6–8,585.9) | 6,135.4 (5,186.9–7,870.2) | −16.69 | −0.67 | −0.91, −0.43 |
| Western Europe | 1,072,161 (763,256–1,230,427) | 458,277 (367,454–696,785) | 4,662.1 (3,318.9–5,350.3) | 2,189.0 (1,755.1–3,328.2) | −53.05 | −2.35 | −2.53, −2.17 |
| Western Sub-Saharan Africa | 4,024,369 (1,915,304–7,643,700) | 8,102,044 (4,170,994–15,016,511) | 11,156.0 (5,309.4–21,189.2) | 9,705.6 (4,996.5–17,988.7) | −13.00 | −0.52 | −0.63, −0.40 |
| DALYs aggregate the 13 ConGD causes (9 structural anomalies + 4 hemoglobinopathies). ASR: age-standardized DALY rate per 100,000 population. EAPC: estimated annual percentage change derived from log-linear regression of ln(ASR) on calendar year, 1990–2023. | | | | | | | |
